# Equus roundworms (*Parascaris univalens*) are undergoing rapid divergence while genes involved in metabolic as well as anthelminic resistance are under positive selection

**DOI:** 10.1186/s12864-022-08702-6

**Published:** 2022-07-04

**Authors:** Lei Han, Tianming Lan, Yaxian Lu, Mengchao Zhou, Haimeng Li, Haorong Lu, Qing Wang, Xiuyun Li, Shan Du, Chunyu Guan, Yong Zhang, Sunil Kumar Sahu, Puyi Qian, Shaofang Zhang, Hongcheng Zhou, Wei Guo, Hongliang Chai, Sibo Wang, Quan Liu, Huan Liu, Zhijun Hou

**Affiliations:** 1 College of Wildlife and Protected Area, Northeast Forestry University, Harbin, 150040 China; 2 BGI Life Science Joint Research Center, Northeast Forestry University, Harbin, China; 3 State Key Laboratory of Agricultural Genomics, BGI-Shenzhen, Shenzhen, 518083 China; 4 College of Life Sciences, University of Chinese Academy of Sciences, Beijing, 100049 China; 5 Guangdong Provincial Key Laboratory of Genome Read and Write, BGI-Shenzhen, Shenzhen, 518120 China; 6 China National GeneBank, BGI-Shenzhen, Shenzhen, 518083 China; 7 Harbin Northern Forest Zoo, Harbin, 150040 China; 8 Inner Mongolia Agriculture University, Hohhot, 010000 China; 9 Center for Animal Disease Control and Prevention of Ordos, Inner Mongolia Ordos, 017000 China; 10 State Key Laboratory of Veterinary Biotechnology, Harbin Veterinary Research Institute, Chinese Academy of Agricultural Sciences, Harbin, 150040 China; 11 Key Laboratory of Wildlife Conservation, China State Forestry Administration, Harbin, 150040 China

**Keywords:** Diversification, *Parascaris univalens*, Adaptation, Evolution

## Abstract

**Background:**

The evolution of parasites is often directly affected by the host's environment. Studies on the evolution of the same parasites in different hosts are of great interest and are highly relevant to our understanding of divergence.

**Methods:**

Here we performed whole-genome sequencing of *Parascaris univalens* from different Equus hosts (horses, zebras and donkeys). Phylogenetic and selection analyses were performed to study the divergence and adaptability of *P. univalens*.

**Results:**

At the genetic level, multiple lines of evidence indicate that *P. univalens* is mainly separated into two clades (horse-derived and zebra & donkey-derived). This divergence began 300–1000 years ago, and we found that most of the key enzymes related to glycolysis were under strong positive selection in zebra & donkey-derived roundworms, whereas the lipid-related metabolic system was under positive selection in horse-derived roundworms, indicating that the adaptive evolution of metabolism has occurred over the past few centuries. In addition, we found that some drug-related genes showed a significantly higher degree of selection in diverse populations.

**Conclusions:**

This work reports the adaptive evolution and divergence trend of *P. univalens* in different hosts for the first time. Its results indicate that the divergence of *P. univalens* is a continuous, dynamic process. Furthermore, the continuous monitoring of the effects of differences in nutritional and drug histories on the rapid evolution of roundworms is conducive to further understanding host-parasite interactions.

**Supplementary Information:**

The online version contains supplementary material available at 10.1186/s12864-022-08702-6.

## Introduction

*Parascaris univalens* is a large parasitic nematode that predominantly infects foals and weanlings. *P. univalens* has a direct lifecycle in which infective eggs ingested from the environment hatch in the horse’s stomach. The larvae then penetrate the intestinal wall and moult by migrating to the liver and lungs and eventually return to the small intestine to develop into adults. Understanding the genetic characteristics of Parascaris worms in different regions or environments is essential for monitoring the development of new variants [1]. Studies on the population genetic structure of *P. univalens* have been conducted in several areas [2, 3]. The Equus species, such as horse (*Equus caballus*), zebra (*Equus zebra*) and donkey (*Equus asinus*), are the reservoir hosts of *P. univalens*. Despite the close relationships of these hosts, the extent of their habitats, food composition, digestion levels, and history of human intervention are very different, and the impact of these factors on large parasites living in these hosts remains unknown. Studying selection and evolutionary processes in different living environments provides insight into the adaptation of *P. univalens*.

Nematodes have evolved to exploit highly diverse ecological niches. Parasitic nematodes have adapted to a wide range of threats, including climatic and nutritional threats, and the immune responses of hosts [4]. These factors represent significant selective pressures imposed on nematodes by the environment throughout their evolutionary history. The hosts of *P. univalens* include a variety of equine species, and the geographical distributions, dietary structures and immune abilities of these hosts are also different. Donkeys have much lower energy and protein requirements than other equids [5], and even the metabolic response during exercise differs depending on the amount of food given to a horse [6], which may also represent a challenge to parasites. Extensive genetic diversity provides the genetic basis for the adaptive evolution of nematodes, but successful evolution will also come at a cost to the host [7]. The genetic diversity of roundworms contributes to their evolutionary adaptation. Understanding these processes is crucial to comprehending the specific evolutionary trends of parasites [8]. The genetic differences between populations can reveal much about the basic evolutionary process driven by changes in the environment [9]. Studying the adaptation of parasites along with different hosts is of great significance for understanding parasitic preferences.

The control of parasitic nematodes feeding on animals relies almost exclusively on anthelmintics, which have proven to be effective in the short-term management, but their long-term effectiveness has been questioned due to the widespread emergence of drug resistance [10]. There is widespread concern about the risk associated with relying on anthelmintics with hundreds of millions of doses of these drugs being donated and used every year [11]. Due to the inconsistency of drug histories in different regions or on different farms, roundworms face varying degrees of selection pressure. Timely monitoring of drug use in relation to the evolution of roundworms is of great significance. In recent years, genome scanning has become an effective means of revealing the genetic determinants of adaptive evolution in different habitats in some organisms. Selected loci exhibit lower polymorphism than other regions of the genome, which enables the formation of highly divergent regions that serve as the genetic foundation for divergence [12, 13]. Here we analysed the genome characteristics of *P. univalens* populations found in three main hosts: horse (*E. caballus*), zebra (*E. zebra*) and donkey (*E. asinus*) in northeastern China. We present the first report on the recent divergence of *P. univalens* populations, and speculate that it might be linked to host digestion and metabolic preferences. In addition, the role of selection pressure in the population was evaluated at the genomic level. This work provides insight into *P. univalens* differentiation and metabolism and drug selection pressure.

## Results

### Genome resequencing and genetic variations

**Fig. 1:**
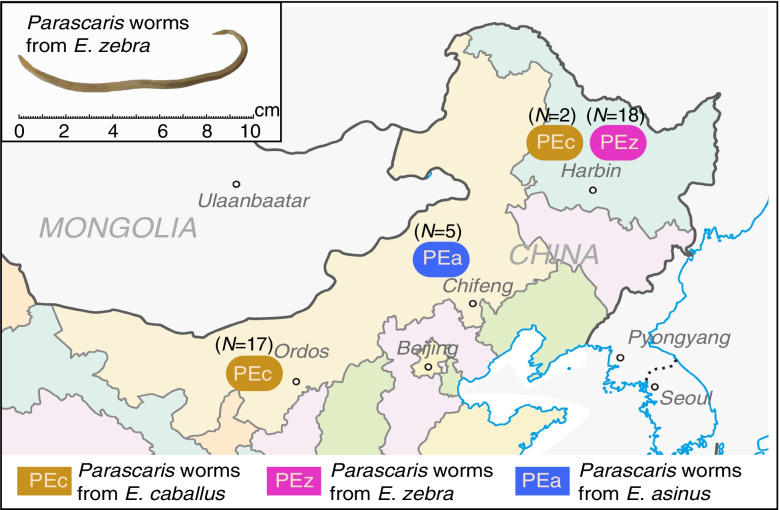
Sampling localities and geographical distribution of the three distinct populations of *P. univalens*. The upper left corner was a zebra-derived roundworm

A total of 42 individuals from three *P. univalens* populations of Equus (PEc, *n* = 19), zebra (PEz, *n* = 18), and donkey (PEa, *n* = 5) hosts from Inner Mongolia and Heilongjiang, China, were performed whole-genome sequencing (Fig. 1, Table S1). We identified 4,398,519 SNPs with a genome-wide distribution of 1 SNP per 57 bp on average (Fig. S1). Genome sequencing was accomplished with an average depth of ~ 20X (Fig. S2) and the average mapping rate was 98.17%. In addition, the genomic coverages were higher than 90% in all individuals (Table S2). Currently, there may be two types of Equus roundworms due to different chromosome numbers (*P. equorum n* = 4; or *P. univalens n* = 2). We randomly selected 1–4 samples from each sampling site for karyotype identification and found that all samples had two chromosomes (see Methods; Fig. S3). The results indicated that the collected samples were all *P. univalens*. In addition, we calculated the observed heterozygosity and the expected heterozygosity, which indicated that the inbreeding coefficient between individuals was very low (average F < 0.1), and the relationships were distant and could represent the whole population (Table S3).

### Genetic differentiation in the *P. univalens*

**Fig. 2:**
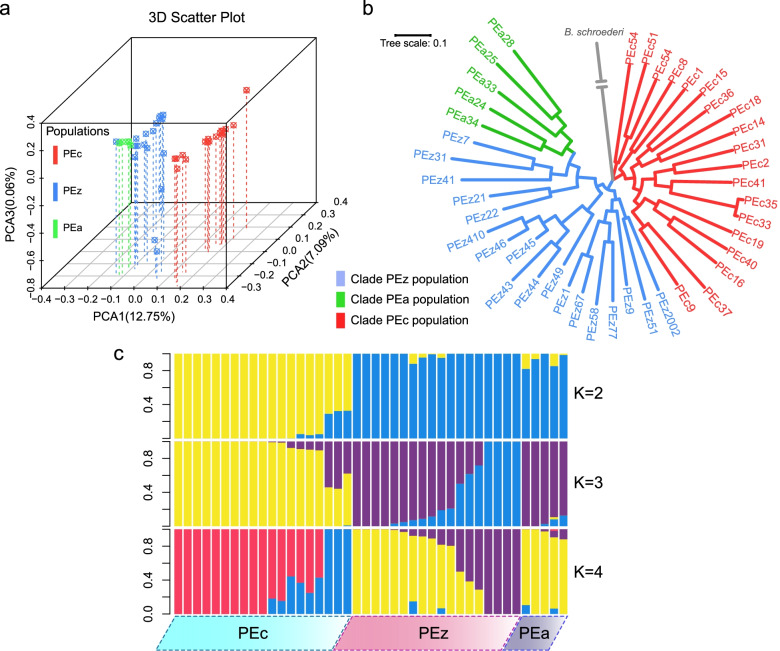
Population structure and relationships of *P. univalens* from horse, zebra and donkey. **a** PCA plots of the first three components. The fraction of the variance explained was 12.75% for PC1, 7.09% for PC2 and 0.06% for PC3; (**b**) Phylogenetic tree (ML tree with 1000 bootstraps) of all samples inferred from whole-genome tag SNPs, with *B. schroederi* as an outgroup; (**c**) Population structure plots with K = 2–4. The y axis quantifies the proportion of the individual’s genome from inferred ancestral populations, and x axis shows the different populations

Principal component analysis (PCA) supported the clear separation among *P. univalens* populations (Fig. 2a), with PC1 and PC2 separating the PEc and PEz&PEa populations (*P* < 0.05). All PCs showed that PEz and PEa were in the same cluster. In addition, the phylogenetic relationships among the three populations inferred based on the ML tree highlighted a similar division to that indicated by PCA (Fig. 2b). The tree showed two distinct clusters, where the PEc population seemed to be a separate clade, while the other two formed a distinct clade. Although the sampling sites of PEc and PEz partially overlapped, the divergence between them was still clear. Population admixture analysis further confirmed the two distinct clusters presented by PCA and ML tree, where PEz and PEa shared more ancestral components in common (Fig. 2c). We scanned the paired identity-by-descent (IBD) regions at the genome-wide level in all individuals and found that PEz and PEa shared more IBD regions (98.5% of PEa shared with PEz), while PEc and the other two populations presented almost no shared large IBD fragments (Fig. S4, Fig. S5). In addition, lower interpopulation *Fst* values were identified in the PEz and PEa populations, also indicating a closer relationship between them (Fig. S5).

### Inference of the demographic history and divergence time of *P. univalens* populations

**Fig. 3:**
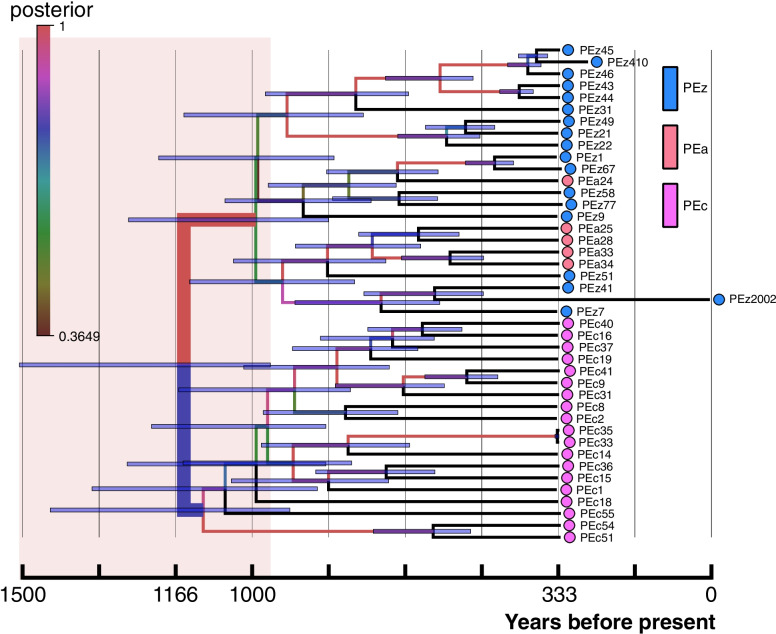
Chronogram of the *P. univalens* based on Bayesian coalescent analysis of SNP data using SNAPP. Nodes with high support (posterior probability = 1.00) are filled in red color. Error bars represent the 95% highest posterior densities (HPD). The colored circles represent different populations

**Fig. 4:**
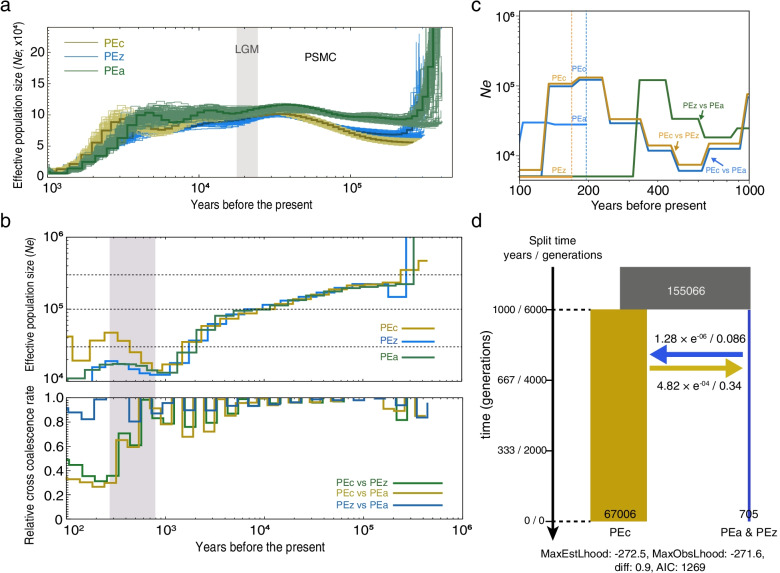
Demographic history of the *P. univalens* populations reconstructed from the reference and population resequencing genomes. **a** The colored lines represent the estimated effective population size of each population. The 100 curves of each color represent the PSMC estimates for 100 sequences randomly resampled from the original sequence. The generation time (*g*) and the neutral mutation rate per generation (*μ*) of *P. univalens* were 0.17 years and 0.9 × 10^−8^, respectively. **b** Coalescent-based inference of demographic history using MSMC2. The upper panel shows the effective population sizes (*Ne*) of three populations, while the lower panel shows the split time between three populations; (**c**) Effective population size and split time based on SMC +  + method. **d** Schematic of demographic scenario modeled in Fastsimcoal2

To examine genome-wide divergence times among *P. univalens* populations, we constructed a molecular clock phylogenetic tree based on the calibrated mutation rate using all SNP sites. The tree topology showed that the divergence time of PEc and PEz&PEa was approximately 900–1500 years ago when the posterior probability was > 95% (Fig. 3). The PSMC results showed similar effective population sizes (*Ne*) of three populations (Fig. 4a). Further Ne estimation inferred by MSMC2 also supported the PSMC result, showing a similar trend before 1000 years ago, indicating the existence of a possible common ancestor (Fig. 4a, b). However, the Ne values of the three populations began to diverge in the last ~ 800 years (Fig. 4b). We found that the PEc population genetically separated from PEz and PEa (Fig. 4b) ~ 300 years ago with the relative cross coalescence rate (RCCR) of less than 0.5. However, there was no obvious sign of separation between PEz and PEa (RCCR > 0.5). The separations among these three populations were further supported and validated by the results inferred by SMC +  + , which were independent of phased genotypes (Fig. 4c). We carefully compared the relationship between the observed separations and the topology of the phylogenetic tree and found similar results. Both results showed that PEc vs. PEz, and PEc vs. PEa presented obvious divergence, but PEz vs. PEa did not show complete differentiation. Although the divergence times estimated based on the above methods were not entirely consistent, they all indicated that the observed differentiation occurred recently. Taken together, we inferred from the results that *P. univalens* was mainly divided into two clades, one was horse-derived (PEc), and the other was zebra & donkey-derived (PEz&PEa). The conservative divergence time was estimated to be within the last 300 years. Finally, we used fastsimcoal2 to evaluate the population size after divergence based on the observed joint site frequency spectrum (SFS; Fig. S6) and found that the PEc population size was significantly larger than PEz&PEa population size after this divergence event (Fig. 4d). The best coalescent simulation model inferred by fastsimcoal2 also indicated early bidirectional gene flow between PEc and PEz&PEa.

### The most possible demographic model in *P. univalens* populations

**Fig. 5:**
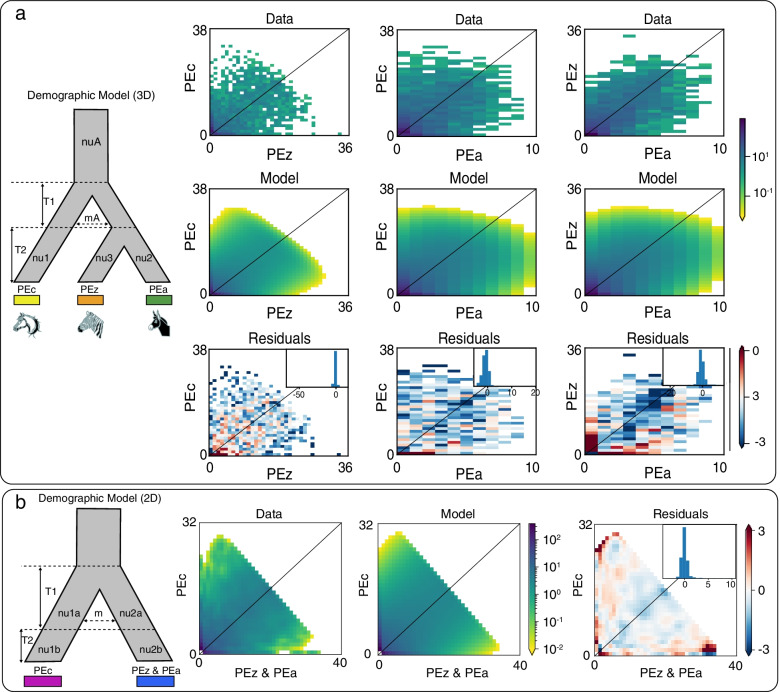
Demographic inferences and early gene flow of *P. univalens* populations. **a** The population genetic model comparison using the three-dimensional site frequency spectrum (3D-SFS) between the PEc, PEz and PEa populations. A simplified graph of the best-fit model is depicted, along with the comparison of the 3D-SFS for data, model and residuals. **b** Results of the population genetic model comparison using the two-dimensional site frequency spectrum (2D-SFS) between PEc and PEz & PEa population along with the 2D-SFS for data, models, and residuals

To better understand the recent divergence of *P. univalens*, we used δaδi to further explore the demographic history of the divergence. Following Portik’s [14] method, we employed a four-round optimization technique to ensure that all final optimizations resulted in a similar log-likelihood score (Table S4). In order to better validate the possible divergence patterns between populations, we first constructed ten 3D models for three independent populations (Fig. S7). The model with the lowest-scoring log-likelihoods was the “Adjacent ancient migration, shorter isolation” model (Fig. 5a, Table S5). This ancient migration model involving early gene flow with symmetric migration was supported as the best fit for the three populations. Next, in our eight 2D simulations (Fig. S8), the ancient migration or secondary contact plus instantaneous size change model involving divergence with ancient continuous symmetrical migration, isolation with instantaneous size change provided the best fit for the PEc and PEz&PEa lineages (Fig. 5b, Table S5). By comparing the best 2D and 3D models, it was found that the best 2D models showed larger log values and lower residuals, which coincided with the results of our phylogenetic tree analysis. Furthermore, the best model revealed a possible divergence of roundworm populations, that is, there was bidirectional gene flow in early ancient periods, but in the middle and current stages, gene flow between the populations almost ceased.

### Selection of metabolism and resistance-related genes in different populations

**Fig. 6:**
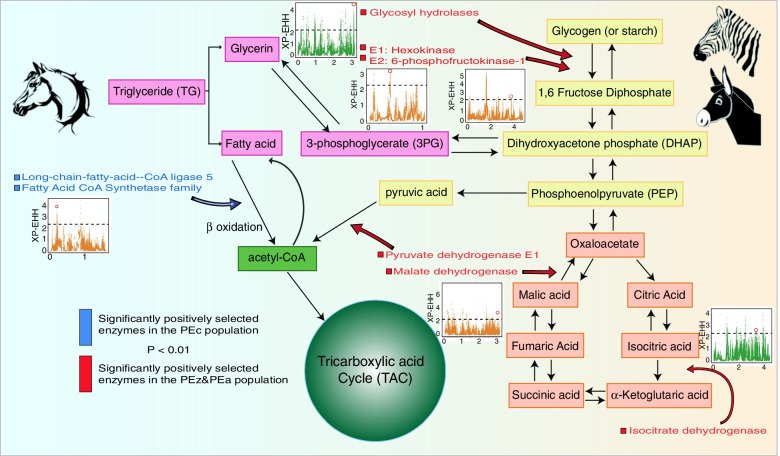
Schematic diagram of glycolysis, tricarboxylic acid cycle, and lipid metabolism. The red arrow represents significantly positively selected enzymes in the PEz&PEa clade (*P* < 0.01), and the blue arrow represents significantly positively selected enzymes in the PEc clade (*P* < 0.01). The Manhattan plot is the XP-EHH score of the 50 k region around the related genes

In-depth genome scanning and functional annotation helped us to understand the observed population divergence. Genome-wide nucleotide diversity (*π*) was computed for each population based on all samples. Additionally, we identified genomic regions as candidate divergent regions (CDRs) among the PEc, PEz and PEa populations (Table S6, Fig. S9). We used iHS to detect genes under recent natural selection in the PEc and PEz&PEa populations. A total of 1,046 SNPs in PEc and 1,093 SNPs in PEz&PEa were identified within the top 1% iHS scores (Fig. S10). These SNPs were annotated to 290 and 254 functional genes, respectively. The GO functional enrichment showed that they were mainly enriched in GO terms such as metabolism and regulation of gene expression (Table S7). The results of KEGG enrichment also showed that the two clades presented significant selection signals in metabolism-related signalling pathways (Fig. S11) [15, 16]. In addition, we used the XP-EHH method to screen genes that may have been positively selected under different environmental pressures by comparing the PEc and PEz&PEa populations. The two-sided *P* value test was used to scan genome regions with selection sweep signals in the two clades. Interestingly, the differences in carbohydrate metabolism and lipid metabolism were extremely significant in the two clades (Fig. S12, Fig. S13, Table S8). The PEz&PEa clade showed significant positive selection on almost all key enzymes involved in glycolysis and the tricarboxylic acid cycle. These loci aroused our interest, and we re-examined the selection dynamics of their surrounding regions (Fig. 6). These regions included includes the kinases (E1:hexokinase and E2:6-phosphofructokinase-1) involved in the two most critical irreversible reactions in the first stage of converting glucose to pyruvate under pyruvate anaerobic conditions. The dehydrogenase (isocitrate dehydrogenase) involved in the irreversible reaction of isocitrate oxidative decarboxylation to α-ketoglutarate was also significantly positively selected (*P* < 0.05). The collective selection of enzymes in the glycolysis process and tricarboxylic acid cycle showed that PEz&PEa presented a greater demand for this process than PEc. In addition, members of the lipid synthase family, which are involved in the uptake of fatty acids, were significantly positively selected in PEc. Parasitic helminths contain appreciable quantities of lipids. However, most intestinal helminths do not utilize significant amounts of lipids even during starvation and under aerobic conditions [17], mainly due to their anaerobic mode of life. The significant selection signals detected among these enzymes, such as members of the fatty acid CoA synthetase family and long-chain fatty acid CoA ligase 5, suggest that they might be also involved in some other processes as well, not just lipid uptake or metabolism.

Current anthelmintics mainly present two modes of action, one mode is characterized by more rapid action on membrane ion channels, and the other is a relatively slow biochemical reaction. These common types of anthelmintics include benzimidazoles (BZs), macrolides (MLs), nicotinic acetylcholine receptor agonists [18], and aminoacetonitrile derivatives (AADs). We screened the main genes related to resistance to all of the abovementioned anthelmintics reported thus far (Table 1). We also scanned iHS scores within 50 kb of all these gene regions and calculated the nucleotide diversity (*π*) and Tajima’s D value of the three populations with 10 kb sliding windows. The results showed that multiple resistance-related genes were under strong positive selection among different populations (Table 1). The genetic diversity of some drug-related gene regions was significantly low and conserved. We identified three classic resistance loci (167/Phe, 198/Glu, and 200/Phe) of the BZ resistance gene β-tubulin [19] and found that none of the individuals in the three populations showed resistance mutations based on sequence alignment (Fig. S15a). However, these gene regions were found to be under strong recent positive selection (Fig. S14c). Selection signals were also identified in other resistance-related genes in the population also discovered (Fig. S15—S17). For example, the multidrug resistance protein *pgp-3* were found to be under strong positive selection in the PEc population. Glutamate-gated chloride channel alpha (*glc-1*), which is related to ivermectin resistance, showed strong positive selection in the PEa populations. Three gene of cytochrome P450 family (CYP_4C1, CYP_4V2, CYP_3A7) showed strong positive selection in the PEc populations. The positive selection of these gene regions differed in the three populations, which may be related to the drug history in different environments. Nevertheless, it was obvious that certain instances of genetic selection were highly conserved and significant in some populations. The results show that the selection of these resistance-related genes will be maintained in independent populations and will continue to be passed down to the next generation. It should be noted that these instances of selection were population specific and have the potential to promote population differentiation.

**Table 1:** Nucleotide diversity (*π*) and Tajima’s D values around the key resistance-related proteins’ region in PEc, PEz and PEa populations

Scaffold ID	Start	End	Description^a^	*π*			*Tajima’D*			Significantly selected populations
				PEc^b^	PEz^c^	PEa^d^	PEc^b^	PEz^c^	PEa^d^	
PgR045	1,372,050	1,381,671	β tubulin	0.0017	0.0019	0.0005	1.4698	2.5324	1.268	PEc, PEz, PEa
PgR004	1,320,231	1,320,527	*glc-1*	0.0021	0.0017	0.0007	-0.4035	2.0857	-1.8797	PEa
PgB04	2,029,204	2,057,834	*pgp-1*	0.0099	0.0095	0.008	1.5276	1.3328	0.0662	-
PgR063X	121,395	124,033	*pgp-3*	0.0002	0.0007	0.0002	0.3278	-0.0274	-1.8733	PEa
PgB05	571,021	589,065	*mrp-7*	0.0119	0.0089	0.0097	-0.2053	1.2363	0.4773	-
PgR035X	1,170,116	1,202,939	*mrp-6*	0.0051	0.0021	0.0047	2.0705	0.7746	0.6784	-
PgB09	815,741	828,043	*mrp-5*	0.0053	0.0037	0.002	0.1012	0.6472	-0.6395	-
PgB10	552,422	565,520	*mrp-4*	0.0093	0.0099	0.0092	0.9641	1.3762	1.5909	-
PgB03	2,066,848	2,079,134	*haf-2*	0.0033	0.0036	0.0047	0.783	1.4183	0.811	-
PgR004	2,938,177	2,953,151	*haf-5*	0.0232	0.02	0.0173	0.7172	1.6294	-0.3418	-
PgR006	879,399	911,228	*cup-4*	0.0083	0.0079	0.0075	2.0797	2.1662	0.6134	-
PgR009X	834,742	840,950	*bre-4*	0.0064	0.002	0.0004	1.2283	1.3452	0.7223	-
PgR004	1,575,226	1,593,120	*unc-29*	0.0031	0.0046	0.0036	0.1117	1.9462	1.3307	-
PgB12X	400,372	407,591	*unc-38*	0.0003	0.0001	0.0004	-1.4946	0.0306	-0.2792	PEc
PgR024	1,427,386	1,436,555	Glycosyl hydrolase 18	0.0049	0.0074	0.002	-0.7514	0.6444	0.2132	-
PgR003	3,165,465	3,176,989	nrf-6	0.0052	0.0051	0.0034	2.4611	3.49	2.2507	-
PgR008	3,213,688	3,247,697	ced-7	0.0082	0.0067	0.0064	0.5927	0.2795	0.3853	-
PgR001X	1,705,437	1,736,423	aex-3	0.0018	0.0005	0.0011	0.2075	2.0238	-0.1421	-
PgB07	1,545,397	1,551,068	CYP_2B19	0.0013	0.0003	0.0018	1.0607	1.0553	0.9086	-
PgR002	512,210	524,078	CYP_13A8	0.0002	0.0014	0.0051	-0.1263	1.1847	0.6614	-
PgR010	2,986,365	2,996,675	CYP_3A31	0.0072	0.0086	0.0065	1.3057	2.4035	2.0071	-
PgR020	456,556	464,491	CYP_2C8	0.0068	0.0081	0.0091	0.7683	1.0655	0.6074	-
PgR027	284,432	291,992	CYP_2C25	0.0036	0.0043	0.0035	0.3752	1.6727	-0.4168	-
PgR033	836,482	846,733	CYP_4C1	0.0015	0.0107	0.0097	-2.6423	2.4022	1.5867	PEc
PgR049	713,990	725,852	CYP_4V2	0.0001	0.0005	0.0008	-1.4324	0.347	0.639	PEc
PgR012	2,339,498	2,343,997	CYP_3A7	0.0063	0.0125	0.0094	-1.2345	1.6788	0.0835	PEc

^a^note: β tubulin, β tubulin; *glc-1*, glutamate-gated chloride channel alpha; *pgp*, multidrug resistance-associated protein family; *mrp*, multidrug resistance protein family; *haf*, half transporter family; *cup-4*, acetylcholine receptor-like protein; *bre-4*, beta-n-acetylgalactosaminyltransferase bre-4; *unc*, Acetylcholine receptor family; *nrf-6*, Nose resistant to fluoxetine protein 6; *ced-7*, ABC transporter ced-7; *aex-3*, MAP kinase-activating death domain protein; *CYP*, cytochrome P450 family

^b^PEc, *P. univalens* from *Equus caballus*

^c^PEz, *P. univalens* from *Equus zebra*

^d^PEa, *P. univalens* from *Equus asinus*

## Discussion

The ecological environment inhabited by parasites differs from that encountered by ordinary animals, and the survival of parasites is more dependent on the intestinal environment. The evolution of a single species is a long and complicated process. Numerous environmental changes and related factors affect this process. Our study used a combination of explicit genetic analysis and demographic models to determine the possibly diversified mechanisms that occur in different intestinal environments. We estimated the population histories of three populations using the PSMC and SMC +  + methods, and their population histories showed exactly the same trend before 10,000 years ago, while differences were found in recent population history estimates. In addition, we applied δaδi 2D/3D models to simulate the possible demographic history. The ancient migration or secondary contact model and the immediate size change model, were found to be effective in explaining the demographic differences and recent divergence of the *P. univalens* populations. In addition, the demographic history showed that *P. univalens* is undergoing divergence. We summarize the main findings regarding the diversification of *P. univalens*, and provide a perspective for the future monitoring of roundworm ecology in a timely manner to address possible unfavourable mutations.

The host's influence on parasite evolution cannot be ignored. A change in the host's diet represents both an opportunity and a challenge for parasites. From the perspective of glycolysis, we found that most of the key enzymes involved in glycolysis were subjected to a higher degree of recent positive selection in PEz and PEa than in domestic horses. Glycolysis and tricarboxylic acid cycle, which represent the most important route whereby roundworms obtain ATP, seem to have "degraded" in domestic horse roundworm populations. Experiments have shown that the contents of fatty acids such as palmitic acid, palmitoleic acid, stearic acid, and oleic acid in a parasite are almost the same as those in its specific host and the changes in the ratio of fatty acids tend to be synchronized between these organisms [20]. Immunological evasion could be a major purpose. Our findings in Equus roundworms supported this notion, which as they showed a strong selection of genes involved in lipid synthesis. In addition to nutrition and maintaining physiological integrity, an obviously more important goal is the potential to maintain consistency with the host’s various fatty acid patterns. The evolution of lipid composition regulation in parasites may influence host suitability [21]. However, we believed that in the case examined in the present study, this consistency is more likely related to intestinal and surrounding lipid deposition in the host, rather than the total lipid ratio.

The issue of drug resistance has been widely mentioned over the past two decades. Parasites show strong adaptability, and roundworms lay more than 200,000 eggs per day [22], almost certainly providing a sufficient mutational basis for resisting environmental changes. The problem of drug resistance has led to significant economic losses in industries such as animal husbandry, and the control of parasites has become an important expenditure [23]. The negative Tajima’s D associated with most genes regarded as selected. Our results showed that some resistance-related genes regions, such as β-tubulin, glc-1, pgp-3, mrp-6, cup-4, nrf-6, and CYP family members, have positive or negative Tajima’s D values, suggesting that they may be selected in different populations. Previous studies have focused on the effects of specific mutation sites, such as the classic β-tubulin gene, on drug resistance. We screened the gene frequencies of these genes and their mutation sites in the population and statistically evaluated the conservation of resistance-related genes at the population level. These genes are related to multiple anthelmintics, which may be related to the deworming history. Over time, the frequency of these genes will increase significantly in the population, and attention should be paid to the impact of this selection on species evolution. In addition to anthelmintics, vaccines offer an attractive alternate control strategy for these parasites [24]. Despite the wealth of available control methods, the responses of these parasites are amazing. When we focus on the issue of drug resistance, we should worry more about the impact of such strong selection on species evolution. Compared with natural selection without human interference, the effect of anthelmintics is undoubtedly more direct and stronger, and the consequences of this strategy are challenging to predict. This suggests that we should be cautious in dealing with the issue of drug resistance and adopt more scientific strategies. It should be noted that *P. univalens* is highly adaptable and evolves quickly. As the gene frequency of certain drug-related genes in specific populations further increases, it is likely that *P. univalens* will differentiate into new subpopulations with specific phenotypes.

This work provides a reference for monitoring the evolution of parasites and elucidating evolution under the action of both natural selection and drugs. Due to the limitation of sample size, some accidental or other environmental factors potentially related to natural selection in *P. univalens* cannot be fully considered. We only considered two types of highly significant factors (metabolism and anthelmintics), and other selection effects still need to be verified in a broader range of *P. univalens* populations in the future.

## Methods

### Sample collection

Seventeen roundworms were collected from six naturally infected horses (*E. caballus*) treated with anthelmintics on a farm located in the Ordos, Inner Mongolia, China. While two horse roundworm individuals were collected after anthelmintic treatment from a farm in Harbin, Heilongjiang, China. Five roundworms from three donkeys (*E. asinus*) were collected after anthelmintic treatment from a farm in Chifeng, Inner Mongolia, China. Eighteen roundworms from ten zebra (*E. zebra*) were obtained after anthelmintics treated from Harbin Northern Forest Zoo, Heilongjiang, China. It is worth noting that at the Harbin Zoo, zebras and domestic horses share the same pasture. Before treatment with anthelmintic, we collected the feces of all zebras and domestic horses in the Zoo to detect the infection of the roundworms. Interestingly, *P. univalens* eggs were only detected in some zebra feces but not in domestic horse feces. All specimens were washed extensively in sterile physiological saline (37 °C), snap-frozen and transported with dry ice, and then stored at -80 °C until further use.

### Karyotyping

We performed karyotyping on the collected samples. Worms were carefully dissected and the gonads were located and excised. The gonads were then processed for karyotyping as previously described [3]. Using a modified freeze-crack method permeated and fixed embryos. Briefly, the embryos were immersed in KCl (0.075 M) hypotonic for 5 min, then rinsed with methanol/acetic (3:1) acid solution. Next, drip 45% acetic acid on the siliconized coverslip. After pressurizing for about 60 s, put the slider in liquid nitrogen and freeze for 1–2 min. Staining was carried out with 4′,6-diamidino-2-phenylindole (DAPI) for 5 min, and the slides were then examined under a fluorescence microscope.

### Nucleic acid isolation, library construction and sequencing

Total genomic DNA was isolated using sodium dodecyl sulphate/proteinase K digestion [25] followed by phenol–chloroform extraction and ethanol precipitation. Genomic DNA was sheared into 200–800 bp for paired-end libraries preparation according to the manufacturer’s instructions of the DIPSEQ platform (BGI-Shenzhen, Shenzhen, China). Libraries were then subjected to the DIPSEQ-T1 sequencer for short-read whole genome sequencing (WGS) sequencing (Table S1).

### Read mapping and SNP calling

High-quality reads were aligned to the *P. univalens* reference genome (WormBase accession ID: GCA_002259215.1) using BWA-MEM (0.7.13-r1126) [26] with default parameters. SAMtools (v0.1.19) [27] was used to convert mapping results into the BAM format and filtered the unmapped and non-unique mapping reads. The *Parascaris equorum* reference genome (WormBase project ID: PRJEB514) was used for species identification. Duplicated reads were marked with the Picard Tools (picard.sourceforge.net, Version: 2.1.1). Then Genome Analysis Toolkit (GATK v 4.0.3.0) [28] was used to population SNP calling. Then hard filtering was applied to the raw variant set using "QD < 2.0 || FS > 60.0 || MQ < 20.0 || MQRankSum < -12.5 || ReadPosRankSum < -8.0" –filter-name "snp_filter". SNPs with > 1% missing data or < 0.01 minor allele frequency (MAF) were filtered out using vcftools (v0.1.12a) [29] for downstream bioinformatic analyses. Variants Annotation of variants was performed according to the reference genome using the package ANNOVAR (Version: 2015–12-14). Using the SAMtools software, the coverage of each sample was counted based on aligned BAM data.

### Diversity analysis

SNP density was counted with a 10 kb sliding window using VCFtools (v0.1.13) software [29]. Genome-wide nucleotide diversity (*π*) and Tajima’s D were computed by sliding windows of 1 kb using all individuals in each population using VCFtools (v0.1.13). The Weir-Cockerham fixation index (*Fst*) was estimated among three populations with genotype data using the VCFtools (v0.1.13).

### Phylogenetic relationship, genetic structure, and admixture

Principal component analysis (PCA) was carried out using EIGENSOFT [30] software based on the SNP dataset, and the population clustering analysis was conducted in PLINK (v1.90b6.10) [31]. We used genome-wide SNPs to construct the maximum likelihood (ML) phylogenetic tree with 1000 bootstraps using iqtree (v1.6.12) [32]. The genome sequence of *Baylisascaris schroederi* was selected as an outgroup. Population structure was analyzed using the ADMIXTURE (v1.3.0) program with a block-relaxation algorithm. To explore the convergence of individuals, we predefined the number of genetic clusters of K from 2 to 4.

We investigated the relationships within *P. univalens* populations in a coalescent framework with SNAPP implemented in BEAST v2.6.3 [33]. We performed two independent runs with a chain length of 10,000,000 generations, sampling every 1,000 generations. We examined convergence using TRACER v1.7.1 and created a maximum clade credibility tree after a burn-in of 20% via TREEANNOTATOR [34]. According to epidemiological investigation, we assumed that the average generation time of *P. univalens*was 0.17 year, and converted the SNAPP analyses into units of real-time using a mutation rate (*μ*) of 9 × 10–9 per generation per site.

### Estimates of the effective population size and divergence time

The pairwise sequentially Markovian coalescent (PSMC) method [35] was used to evaluate the dynamic change of effective population size (*Ne*) of each population. We used 0.17 year per generation (*g*) and a mutation rate (*μ*) of 9 × 10–9 per generation per site to rescale the time to year [36, 37]. More recent (within 1000 years) changes in effective population size of each population and separation time between different populations were further estimated by using the multiple sequentially Markovian coalescent (MSMC2) [38], which can much compensate for results from PSMC. However, the inference accuracy of MSMC2 largely depends on the phasing accuracy of genotypes. Switch error rates will introduce bias in the calculation. To further confirm results from MSMC2, we also used Sequentially Markovian Coalescent (SMC + +) methods [39] to do the same analysis as MSMC2. The SMC +  + used phasing-free genotype data to do the population history and separation time inference, which become a reliable method to support inferences from MSMC2. For SMC +  + , we set the upper bound for the number of generations to 10,000 to estimate size history and calculate the lower bound based on a heuristic approach. For MSMC2, we first phased all SNPs of each individual by using beagle (v5.0) [40], then the calculation was performed with the following parameters: -i 20 -t 6 -p '10*1 + 15*2'. The mutation rate (*μ*) of *P. univalens* for SMC +  + and MSMC2 have used the same values as for PSMC.

### Demographic inference using fastsimcoal2 and δaδi

We used the fastsimcoal2 [41] approach to deduce the recent demographic history of *P. univalens* populations. We chose only SNPs located in intergenic regions to avoid the influence of SNPs under selection [42]. We used δaδi [43] to investigate alternative demographic scenarios for the species complex. In the absence of historical evidence, we hypothesized that there may or may not be any form of gene flow between roundworm populations. In order to get the best model, we first simulated a total of ten δaδi 3D models, including one simple model, three simultaneous splitting models, one ancient migration model, one simultaneous splitting variations model, one admixed (“Hybrid”) origin model and three divergences with gene flow variations models. When fitting demographic models, we perform multiple runs (100 rounds) and ensure that final optimizations converge on a similar log-likelihood score. The derivative-based BFGS algorithm was used to optimize the composite log-likelihood to estimate demographic parameters. All models and scripts are available at https://github.com/dportik/dadi_pipeline.

### Recent nature selection analysis

Extended Haplotype Homozygosity (EHH) and Integrated Haplotype Score (iHS) methods were used for detecting SNPs under a recently positive selection of three roundworm populations [44]. We use SNPs with an iHS score of top 0.5% and the distance between adjacent SNPs < 50 kb as candidate SNPs [45]. We searched for genes in the 5-kb flanking region from both sides of candidate SNPs and calculated the accumulated iHS scores by adding all iHS scores of candidate genes. Next, to uncover genetic variants under strong positive selection in each host population, we used cross population extended haplotype homozygosity (XP-EHH) method on each pair of combinations (PEc vs PEz, PEc vs PEa and PEz vs PEa) to find population-specific SNPs under strong positive selection. XP-EHH we used in this study was from the R package rehh (v3.1.2; https://cran.r-project.org/web/packages/rehh/vignettes/rehh.html). The regions with *P* values < 0.01 were considered significant signals in the population of interest.

## Conclusion

*P. univalens* is the main parasitic pathogen that infects equine, and it is also the chief culprit in horses' weight loss and weakened immunity. The genetic variation and host differences complicate the development of broad-spectrum diagnostics, therapeutic. Here we report the recent divergence of *P. univalens* and reveal that the rapid evolution of glycolysis-related genes drove this divergence. It is also a key factor leading to the parasitic preference of roundworm populations. In addition, we found that resistance-related genes have a similar tendency, which was the potential impact of overuse of anthelmintics. We have established a rapid evolution gene set of *P. univalens*, which will help managers decide on therapeutic strategies targeting specific populations and allow researchers to monitor the ongoing evolution and diversification of *P. univalens*.

## Supplementary Information

Additional file 1: **Table S1.** Sequencing data. **Table S2.** Summary of sequencing data. **Table S3.** Estimates of expected heterozygosity and observed heterozygosity. **Table S4.** Four rounds of increasingly focused optimizations used in δaδi. **Table S5.** Maximum-likelihood parameter estimates obtained from the joint demographic inference analysis 2D and 3D. **Table S6.** Genomic regions identified as candidate divergent regions. **Table S7.** GO enrichment (Top 20) of iHS significant selection sites in PEc and PEz&PEa clades. **Table S8.** KEGG enrichment of XP_EHH significant selection sites in PEc and PEz&PEa clades. **Figure S1.** SNPdensity. **Figure S2.** Sequencing depth. **Figure S3.** Karyotyping. **Figure S4.** The mapping ratio and shared SNPs distribution. **Figure S5.** Shared identity-by-descent (IBD) region among PEa, PEc and PEz populations. **Figure S6.** The shared IBD and paired Fst values of the three populations. **Figure S7.** Observed SFS between PEc and PEz & PEa clades. **Figure S8.** 3D divergence model used in δaδi. **Figure S9.** 2D divergence model used in δaδi. **Figure S10.** Fst distribution of PEc vs PEa & PEz. **Figure S11.** The iHS score distribution of PEc, PEa and PEz populations. **Figure S12.** KEGG enrichment of iHS significant selection locis of PEc and PEa & PEz respectively. **Figure S13.** GO enrichment of XP_EHH analysis of PEa and PEc & PEz respectively. **Figure S14.** Network diagram of GO function enrichment obtained by XPEHH analysis of PEa and PEc & PEz clades. **Figure S15.** Detection of mutations in three drug-resistant sites of β-tubulin. **Figure S16.** The π distribution and tajima’D distribution in the pgp-3 regions. **Figure S17.** The π distribution and tajima’D distribution in the unc-38, nrf-6, glc-1 and cup-4 regions. **Figure S18.** The π distribution and tajima’D distribution in the CYP3A31, CYP4C1, mrp-1 and CYP4V2 regions.

## Data Availability

The data that support the findings of this study have been deposited into CNGB Sequence Archive (CNSA) [47] of China National GeneBank DataBase (CNGBdb) [48] with accession number CNP0001875. All in-house scripts and codes used in this study were available in the github database (https://github.com/HanLei12321/Equus_roundworms).
