# Supplementary material for: Equus roundworms (Parascaris univalens) are undergoing rapid divergence while genes involved in metabolic as well as anthelminic resistance are under positive selection
Source: BMC Genomics. 2022 Jul 4;23:489. doi: 10.1186/s12864-022-08702-6 (PMC9252044; doi:10.1186/s12864-022-08702-6)
Supplement: Supplementary file 1 — Additional file 1: Table S1. Sequencing data. Table S2. Summary of sequencing data. Table S3. Estimates of expected heterozygosity and observed heterozygosity. Table S4. Four rounds of increasingly focused optimizations used in δaδi. Table S5. Maximum-likelihood parameter estimates obtained from the joint demographic inference analysis 2D and 3D. Table S6. Genomic regions identified as candidate divergent regions. Table S7. GO enrichment (Top 20) of iHS significant selection sites in PEc and PEz&PEa clades. Table S8. KEGG enrichment of XP_EHH significant selection sites in PEc and PEz&PEa clades. Figure S1. SNPdensity. Figure S2. Sequencing depth. Figure S3. Karyotyping. Figure S4. The mapping ratio and shared SNPs distribution. Figure S5. Shared identity-by-descent (IBD) region among PEa, PEc and PEz populations. Figure S6. The shared IBD and paired Fst values of the three populations. Figure S7. Observed SFS between PEc and PEz & PEa clades. Figure S8. 3D divergence model used in δaδi. Figure S9. 2D divergence model used in δaδi. Figure S10. Fst distribution of PEc vs PEa & PEz. Figure S11. The iHS score distribution of PEc, PEa and PEz populations. Figure S12. KEGG enrichment of iHS significant selection locis of PEc and PEa & PEz respectively. Figure S13. GO enrichment of XP_EHH analysis of PEa and PEc & PEz respectively. Figure S14. Network diagram of GO function enrichment obtained by XPEHH analysis of PEa and PEc & PEz clades. Figure S15. Detection of mutations in three drug-resistant sites of β-tubulin. Figure S16. The π distribution and tajima’D distribution in the pgp-3 regions. Figure S17. The π distribution and tajima’D distribution in the unc-38, nrf-6, glc-1 and cup-4 regions. Figure S18. The π distribution and tajima’D distribution in the CYP3A31, CYP4C1, mrp-1 and CYP4V2 regions. [file 12864_2022_8702_MOESM1_ESM.docx]

Supplementary Materials for

**• Equus roundworms (*Parascaris univalens*) are undergoing rapid divergence while genes involved in metabolic as well as anthelminic resistance are under positive selection**

Lei Han, Tianming Lan, Yaxian Lu, Mengchao Zhou, Haimeng Li, Haorong Lu, Qing Wang, Xiuyun Li, Shan Du, Chunyu Guan, Yong Zhang, Sunil Kumar Sahu, Puyi Qian, Shaofang Zhang, Hongcheng Zhou, Wei Guo, Hongliang Chai, Sibo Wang, Quan Liu, Huan Liu, Zhijun Hou

Corresponding author. Email: houzhijundb@163.com; liuhuan@genomics.cn; [lantianming@genomics.cn](mailto:lantianming@genomics.cn); liuquan1973@hotmail.com

**Supplementary Tables**

1. Table S1 - Sequencing data
2. Table S2 - Summary of sequencing data
3. Table S3 - Estimates of expected heterozygosity and observed heterozygosity
4. Table S4 - Four rounds of increasingly focused optimizations used in δaδi
5. Table S5 - Maximum-likelihood parameter estimates obtained from the joint demographic inference analysis 2D and 3D
6. Table S6 - Genomic regions identified as candidate divergent regions
7. Table S7 - GO enrichment (Top 20) of iHS significant selection sites in PEc and PEz&PEa clades
8. Table S8 - KEGG enrichment of XP_EHH significant selection sites in PEc and PEz&PEa clades

**Supplementary Figures**

1. Figure S1 - SNPdensity
2. Figure S2 *-* Sequencing depth
3. Figure S3 - Karyotyping
4. Figure S4 - The mapping ratio and shared SNPs distribution
5. Figure S5 - Shared identity-by-descent (IBD) region among PEa, PEc and PEz populations
6. Figure S6 - The shared IBD and paired *Fst* values of the three populations
7. Figure S7 - Observed SFS between PEc and PEz & PEa clades
8. Figure S8 - 3D divergence model used in δaδi
9. Figure S9 - 2D divergence model used in δaδi
10. Figure S10 - *Fst* distribution of PEc vs PEa & PEz
11. Figure S11 - The iHS score distribution of PEc, PEa and PEz populations
12. Figure S12 - KEGG enrichment of iHS significant selection locis of PEc and PEa & PEz respectively
13. Figure S13 - GO enrichment of XP_EHH analysis of PEa and PEc & PEz respectively
14. Figure S14 - Network diagram of GO function enrichment obtained by XPEHH analysis of PEa and PEc & PEz clades
15. Figure S15 - Detection of mutations in three drug-resistant sites of β-tubulin
16. Figure S16 - The *π* distribution and tajima’D distribution in the *pgp-3* regions
17. Figure S17 - The *π* distribution and *tajima’D* distribution in the *unc-38*, *nrf-6*, *glc-1* and *cup-4* regions
18. Figure S18 - The *π* distribution and tajima’D distribution in the *CYP3A31*, *CYP4C1*, *mrp-1* and *CYP4V2* regions

**Table S1** Sequencing data used for *P. univalens* populations.

|  | Sequencing  platform | Raw data (Gb) | coverage (X) |
| --- | --- | --- | --- |
| WGS (44 samples) | DNBSEQ-T1 | ~5.4 × 44 | Average: ~20 |

**Table S2** Summary of sequencing data of all samples.

| Samples | Map ratio | Mapped Length | Genome | Total Base Number | Sequencing |
| --- | --- | --- | --- | --- | --- |
|  |  |  | Coverage |  | Depth |
| PEc8 | 98.06 | 242939043 | 0.924623 | 7379191103 | 28.0851 |
| PEc35 | 99.5 | 243005286 | 0.924875 | 9660443672 | 36.7675 |
| PEc31 | 99.67 | 242603209 | 0.923345 | 6463458930 | 24.5998 |
| PEc40 | 98.94 | 241063989 | 0.917486 | 3291407118 | 12.5271 |
| PEc2 | 96.31 | 243181907 | 0.925547 | 10307911619 | 39.2318 |
| PEc19 | 99.72 | 242305463 | 0.922211 | 4538632266 | 17.274 |
| PEc55 | 97.73 | 241300242 | 0.918386 | 3498495752 | 13.3152 |
| PEc37 | 97.86 | 241248856 | 0.91819 | 5051469951 | 19.2258 |
| PEc15 | 95.37 | 242793475 | 0.924069 | 5471281685 | 20.8236 |
| PEc51 | 94.67 | 241461752 | 0.919 | 3952951607 | 15.0449 |
| PEc33 | 98.46 | 242524982 | 0.923047 | 6216298886 | 23.6592 |
| PEc1 | 93.9 | 242715410 | 0.923772 | 6500114364 | 24.7393 |
| PEc9 | 95.59 | 243253965 | 0.925821 | 5368055141 | 20.4307 |
| PEc18 | 97.43 | 241730291 | 0.920022 | 3892908745 | 14.8164 |
| PEc54 | 95.26 | 241321158 | 0.918465 | 3542201759 | 13.4816 |
| PEc36 | 99.69 | 242637039 | 0.923473 | 6468187825 | 24.6178 |
| PEc14 | 98.96 | 242757776 | 0.923933 | 6743570299 | 25.6659 |
| PEc41 | 94.67 | 242603716 | 0.923347 | 6506319256 | 24.763 |
| PEc16 | 98.36 | 241299919 | 0.918384 | 3259468966 | 12.4055 |
| PEz22 | 99.54 | 240886927 | 0.916812 | 3657342910 | 13.9198 |
| PEz31 | 99.53 | 242954049 | 0.92468 | 10613902328 | 40.3964 |
| PEz2002 | 98.87 | 239079701 | 0.909934 | 2160977871 | 8.22465 |
| PEz44 | 96.88 | 241686392 | 0.919855 | 5034378816 | 19.1608 |
| PEz51 | 96.3 | 243094953 | 0.925216 | 9866269911 | 37.5509 |
| PEz77 | 99.68 | 241584614 | 0.919468 | 3744986382 | 14.2534 |
| PEz46 | 99.38 | 240569563 | 0.915605 | 2965641622 | 11.2872 |
| PEz41 | 95.2 | 243015007 | 0.924912 | 8070319092 | 30.7155 |
| PEz410 | 99.74 | 243016021 | 0.924916 | 11159801482 | 42.4741 |
| PEz67 | 99.66 | 241057921 | 0.917463 | 3289862672 | 12.5212 |
| PEz49 | 99.2 | 241798652 | 0.920282 | 4852035556 | 18.4668 |
| PEz58 | 99.37 | 239821070 | 0.912756 | 2557394655 | 9.73341 |
| PEz45 | 99.31 | 242115894 | 0.92149 | 5779447504 | 21.9965 |
| PEz9 | 99.83 | 241400268 | 0.918766 | 3542887397 | 13.4842 |
| PEz1 | 99.4 | 241574409 | 0.919429 | 3724282343 | 14.1746 |
| PEz43 | 99.26 | 242179370 | 0.921731 | 5389356615 | 20.5118 |
| PEz7 | 99.53 | 242069922 | 0.921315 | 4894423856 | 18.6281 |
| PEa28 | 98.71 | 241669848 | 0.919792 | 4196242910 | 15.9708 |
| PEa33 | 98.6 | 242528784 | 0.923061 | 6524840829 | 24.8335 |
| PEa24 | 98.31 | 241730658 | 0.920024 | 4272115519 | 16.2596 |
| PEa25 | 99.2 | 240612736 | 0.915769 | 3123987980 | 11.8899 |
| PEa34 | 99.38 | 241041915 | 0.917402 | 3480956061 | 13.2485 |

**Table S3** Estimates of expected heterozygosity and observed heterozygosity of all individuals in this study.

| ID | observed heterozygosity (HOM) | expected heterozygosity (HOM) | N SITES | inbreeding coefficient (*F*) |
| --- | --- | --- | --- | --- |
| PEa24 | 2362551 | 2416978 | 3179889 | -0.07134 |
| PEa25 | 2426727 | 2413281 | 3174635 | 0.01766 |
| PEa28 | 2380409 | 2416666 | 3179440 | -0.04753 |
| PEa33 | 2409400 | 2417248 | 3180316 | -0.01028 |
| PEa34 | 2406611 | 2414374 | 3176096 | -0.01019 |
| PEc1 | 2455638 | 2417130 | 3180152 | 0.05047 |
| PEc14 | 2453819 | 2416982 | 3179979 | 0.04828 |
| PEc15 | 2404701 | 2417280 | 3180375 | -0.01648 |
| PEc16 | 2478428 | 2415316 | 3177491 | 0.08281 |
| PEc18 | 2489762 | 2415957 | 3178476 | 0.09679 |
| PEc19 | 2411363 | 2417004 | 3179929 | -0.00739 |
| PEc2 | 2492557 | 2417274 | 3180357 | 0.09866 |
| PEc31 | 2461873 | 2417296 | 3180404 | 0.05842 |
| PEc33 | 2483245 | 2417509 | 3180678 | 0.08614 |
| PEc35 | 2485215 | 2417568 | 3180754 | 0.08864 |
| PEc36 | 2493935 | 2417101 | 3180076 | 0.1007 |
| PEc37 | 2479863 | 2415901 | 3178418 | 0.08388 |
| PEc40 | 2488701 | 2415316 | 3177478 | 0.09628 |
| PEc41 | 2479002 | 2416833 | 3179809 | 0.08148 |
| PEc51 | 2371335 | 2415781 | 3178230 | -0.05829 |
| PEc54 | 2422355 | 2415467 | 3177797 | 0.00904 |
| PEc55 | 2447710 | 2415418 | 3177702 | 0.04236 |
| PEc8 | 2463603 | 2416896 | 3179866 | 0.06122 |
| PEc9 | 2010315 | 2417716 | 3180884 | -0.53383 |
| PEz1 | 2458139 | 2416242 | 3178797 | 0.05494 |
| PEz2002 | 2510895 | 2404938 | 3162154 | 0.13993 |
| PEz21 | 2442735 | 2414302 | 3175906 | 0.03733 |
| PEz22 | 2441096 | 2416308 | 3178870 | 0.03251 |
| PEz31 | 2535115 | 2417097 | 3180110 | 0.15467 |
| PEz41 | 2437046 | 2417088 | 3180136 | 0.02616 |
| PEz410 | 2492239 | 2417565 | 3180747 | 0.09785 |
| PEz43 | 2500404 | 2417321 | 3180413 | 0.10888 |
| PEz44 | 2581733 | 2417068 | 3180050 | 0.21582 |
| PEz45 | 2493310 | 2417233 | 3180289 | 0.0997 |
| PEz46 | 2517975 | 2414748 | 3176463 | 0.13552 |
| PEz49 | 2485880 | 2417281 | 3180298 | 0.08991 |
| PEz51 | 2519051 | 2417071 | 3180084 | 0.13365 |
| PEz58 | 2537745 | 2409812 | 3169303 | 0.16845 |
| PEz67 | 2455407 | 2414795 | 3176610 | 0.05331 |
| PEz7 | 2432535 | 2416829 | 3179699 | 0.02059 |
| PEz77 | 2487031 | 2416071 | 3178636 | 0.09305 |
| PEz9 | 2476484 | 2415953 | 3178364 | 0.07939 |
| Average | 2453903 | 2416000 | 3178480 | - |

**Table S4** Four rounds of increasingly focused optimizations used in δaδi, and the values of the arguments across rounds.

| Argument | Round 1 | Round 2 | Round 3 | Round 4 |
| --- | --- | --- | --- | --- |
| reps | 10 | 20 | 30 | 40 |
| maxiter | 3 | 5 | 10 | 15 |
| fold | 3 | 2 | 2 | 1 |

**Table S5** Maximum-likelihood parameter estimates obtained from the joint demographic inference analysis 2D and 3D

| **3D Model** | | | | | | | | | | | | |
| --- | --- | --- | --- | --- | --- | --- | --- | --- | --- | --- | --- | --- |
|  | Replicate | log-likelihood | AIC | chi-squared | theta | nu1 | nuA | nu2 | nu3 | mA | T1 | T2 |
| ancmig_  adj_2 | Round_4_  Replicate_4 | -3414.73 | 6843.46 | 65320.56 | 53.59 | 7.67 | 5.40 | 1.09 | 4.76 | 0.52 | 29.91 | 0.05 |
| **2D Model** | | | | | | | | | | | | |
|  | Replicate | log-likelihood | AIC | chi-squared | theta | nu1a | nu2a | nu1b | nu2b | m | T1 | T2 |
| anc_sym  _mig_size | Round_4_  Replicate_5 | -1318.53 | 2651.06 | 1614.23 | 170.66 | 5.79 | 1.26 | 0.96 | 0.47 | 9.26 | 16.04 | 0.04 |

**Table S6** Genomic regions (top 100) identified as candidate divergent regions (CDRs) among PEc, PEz and PEa. Max Fst refers to the maximal windowed Fst value of the CDRs. The ln ratio refers to the maximal π ln ratio value of the CDRs.

| **No.** | **Scaff.** | **Start（bp）** | **End（bp）** | ***Fst* (PEc-PEz)** | ***Fst* (PEc-PEa)** | ***Fst* (PEz-PEa)** | **ln ratio (θ_π,PEc_/θ_π，PEz_)** | **ln ratio (θ_π,PEc_/θ_π，PEa_)** | **ln ratio (θ_π,PEz_/θ_π，PEa_)** | | **Candidate genes** |
| --- | --- | --- | --- | --- | --- | --- | --- | --- | --- | --- | --- |
| 1 | PgR001X | 1870001 | 1880000 | 0.7109 | 0.2027 | 0.4392 | 0.1985 | 0.4211 | 0.2226 |  | |
| 2 | PgB02X | 2140001 | 2150000 | 0.6654 | 0.4946 | -0.0074 | 0.8591 | -0.0368 | -0.8959 | HHATL | |
| 3 | PgB02X | 2870001 | 2880000 | 0.6509 | 0.4488 | 0.059 | 0.0841 | -0.5328 | -0.6169 | VhaA | |
| 4 | PgB02X | 3460001 | 3470000 | 0.6361 | 0.22 | 0.2243 | 1.3601 | 0.1237 | -1.2364 |  | |
| 5 | PgR001X | 1840001 | 1850000 | 0.6041 | 0.1609 | 0.2974 | -0.3601 | -0.8441 | -0.4839 | MTTP | |
| 6 | PgR009X | 3020001 | 3030000 | 0.5946 | 0.0315 | 0.5918 | 2.6068 | -0.016 | -2.6228 | rhy-1 | |
| 7 | PgB02X | 2130001 | 2140000 | 0.5761 | 0.3711 | 0.019 | 1.0579 | 0.5289 | -0.5291 |  | |
| 8 | PgR035X | 1450001 | 1460000 | 0.5729 | 0.4923 | -0.0707 | 2.4802 | 3.448 | 0.9678 | Ctsf | |
| 9 | PgR009X | 2990001 | 3000000 | 0.5659 | 0.0582 | 0.4514 | 1.6174 | 0.182 | -1.4354 | rhy-1 | |
| 10 | PgR004 | 3540001 | 3550000 | 0.5641 | 0.5162 | -0.0487 | 0.0602 | 0.0418 | -0.0183 |  | |
| 11 | PgB07 | 1120001 | 1130000 | 0.5594 | 0.0624 | 0.2995 | 0.7811 | -0.2528 | -1.0339 | attf-4 | |
| 12 | PgR028 | 310001 | 320000 | 0.5573 | 0.6361 | 0.0194 | 0.4948 | 3.6939 | 3.1991 |  | |
| 13 | PgR035X | 1690001 | 1700000 | 0.557 | 0.5114 | -0.1219 | 1.2159 | 1.1012 | -0.1146 | T09B9.5 | |
| 14 | PgB02X | 2850001 | 2860000 | 0.5492 | 0.3765 | 0.015 | 0.1618 | -0.1346 | -0.2963 |  | |
| 15 | PgB02X | 770001 | 780000 | 0.5417 | 0.451 | -0.0089 | 1.108 | 0.5947 | -0.5133 |  | |
| 16 | PgR035X | 510001 | 520000 | 0.5339 | 0.3074 | 0.0579 | -0.236 | -1.2834 | -1.0474 | ANP32A | |
| 17 | PgB02X | 600001 | 610000 | 0.5272 | 0.4639 | -0.0637 | 0.9591 | 1.1191 | 0.16 | PHYHD1 | |
| 18 | PgR007 | 10001 | 20000 | 0.5269 | 0.4751 | -0.0044 | 1.8481 | 1.9774 | 0.1293 | ATP6V1A | |
| 19 | PgR002 | 2350001 | 2360000 | 0.5193 | 0.0341 | 0.3182 | -0.5419 | 0.0221 | 0.564 |  | |
| 20 | PgB02X | 2980001 | 2990000 | 0.5189 | 0.458 | 0.0099 | 1.4813 | 1.3935 | -0.0878 | FASN | |
| 21 | PgB02X | 610001 | 620000 | 0.511 | 0.4547 | -0.0446 | 1.2035 | 1.367 | 0.1635 | cntn5 | |
| 22 | PgR028 | 280001 | 290000 | 0.5102 | 0.6125 | 0.0077 | 0.3213 | 4.5174 | 4.196 |  | |
| 23 | PgB02X | 590001 | 600000 | 0.5095 | 0.3913 | -0.0461 | 2.3947 | 2.4264 | 0.0318 | ARRDC3 | |
| 24 | PgR035X | 1660001 | 1670000 | 0.5081 | 0.5661 | 0.0002 | 0.7496 | 1.1549 | 0.4053 | Dennd2a | |
| 25 | PgR009X | 2590001 | 2600000 | 0.5038 | 0.082 | 0.1932 | -0.075 | -0.3566 | -0.2816 | Asic4 | |
| 26 | PgR004 | 3520001 | 3530000 | 0.5032 | 0.5263 | -0.0557 | -0.0733 | 0.8717 | 0.945 |  | |
| 27 | PgR002 | 2330001 | 2340000 | 0.4991 | 0.2274 | 0.1171 | 0.0657 | 0.0998 | 0.0341 | ARRDC3 | |
| 28 | PgR001X | 800001 | 810000 | 0.4951 | 0.2103 | 0.0696 | 2.5168 | 1.3246 | -1.1921 | Etnk1 | |
| 29 | PgR002 | 2340001 | 2350000 | 0.4865 | 0.0931 | 0.2378 | -0.5309 | -0.1405 | 0.3904 | Plb1 | |
| 30 | PgB02X | 670001 | 680000 | 0.48 | 0.4168 | 0.0007 | 1.38 | 0.7793 | -0.6007 | mek-1 | |
| 31 | PgB07 | 1110001 | 1120000 | 0.4794 | 0.2239 | 0.0421 | 1.1652 | 0.0721 | -1.0932 |  | |
| 32 | PgR063X | 220001 | 230000 | 0.4786 | 0.0446 | 0.2261 | 0.9485 | 0.1355 | -0.8131 |  | |
| 33 | PgR063X | 230001 | 240000 | 0.4719 | 0.0641 | 0.1947 | 0.7112 | 0.0363 | -0.6749 | ARMC9 | |
| 34 | PgR001X | 1410001 | 1420000 | 0.4712 | 0.3903 | 0.0581 | 0.7865 | 0.1148 | -0.6717 | SSRP1 | |
| 35 | PgR050X | 1060001 | 1070000 | 0.4698 | 0.3783 | -0.067 | 2.0565 | 2.4994 | 0.4429 | CBSL | |
| 36 | PgB02X | 2830001 | 2840000 | 0.469 | 0.303 | -0.0009 | 0.7349 | -0.088 | -0.8229 |  | |
| 37 | PgB02X | 720001 | 730000 | 0.4684 | 0.4317 | -0.0965 | 0.706 | 1.2225 | 0.5165 |  | |
| 38 | PgR035X | 1720001 | 1730000 | 0.4664 | 0.4844 | -0.1028 | 0.5939 | 1.0238 | 0.43 | GABBR1 | |
| 39 | PgB02X | 330001 | 340000 | 0.4662 | -0.0557 | 0.3627 | 1.0163 | 0.2381 | -0.7781 |  | |
| 40 | PgR001X | 1520001 | 1530000 | 0.4649 | 0.2884 | -0.0307 | 1.6695 | 0.7469 | -0.9225 | HNRNPA1，FBXL17 | |
| 41 | PgR035X | 1710001 | 1720000 | 0.4622 | 0.4594 | -0.0869 | 0.7617 | 1.346 | 0.5844 | Gabbr1 | |
| 42 | PgR063X | 200001 | 210000 | 0.462 | 0.0403 | 0.1892 | 0.4306 | 0.044 | -0.3866 |  | |
| 43 | PgR009X | 3180001 | 3190000 | 0.4605 | 0.4817 | 0.0525 | 0.2024 | 0.0281 | -0.1744 | Pik3c3 | |
| 44 | PgR035X | 1740001 | 1750000 | 0.4594 | 0.4778 | -0.1112 | 0.8681 | 0.8183 | -0.0498 |  | |
| 45 | PgB07 | 1520001 | 1530000 | 0.4592 | 0.1572 | 0.0655 | 0.4291 | 0.4837 | 0.0545 | CNN1 | |
| 46 | PgR009X | 1480001 | 1490000 | 0.4577 | 0.2764 | 0.1249 | 0.3557 | -0.6611 | -1.0167 | Grm1 | |
| 47 | PgR009X | 3450001 | 3460000 | 0.4529 | 0.3808 | 0.0205 | 1.4049 | 1.3476 | -0.0573 | B3GAT3 | |
| 48 | PgB02X | 3470001 | 3480000 | 0.4506 | 0.124 | 0.181 | 1.9388 | 0.4861 | -1.4527 | F13H6.3，Bche | |
| 49 | PgR035X | 1440001 | 1450000 | 0.4488 | 0.348 | -0.0783 | 2.1105 | 1.9546 | -0.1559 | Dnajb4 | |
| 50 | PgR035X | 1750001 | 1760000 | 0.444 | 0.4724 | -0.0977 | 0.9683 | 0.5416 | -0.4267 |  | |
| 51 | PgR023 | 1680001 | 1690000 | 0.4391 | 0.5662 | -0.0315 | -0.2945 | 0.3111 | 0.6056 | inx-11 | |
| 52 | PgR023 | 200001 | 210000 | 0.4388 | 0.2472 | -0.0162 | 1.7276 | 0.4268 | -1.3009 | Prss30，CTSL | |
| 53 | PgB07 | 1130001 | 1140000 | 0.4369 | 0.0543 | 0.1524 | 0.818 | -0.2329 | -1.0509 | faeA | |
| 54 | PgB02X | 1210001 | 1220000 | 0.4352 | 0.3003 | -0.0476 | 1.1219 | 0.3689 | -0.753 | MME | |
| 55 | PgR028 | 80001 | 90000 | 0.4343 | 0.2158 | 0.1303 | -0.3728 | -0.2946 | 0.0782 | CACNA1C | |
| 56 | PgR028 | 160001 | 170000 | 0.4334 | 0.2176 | 0.141 | -0.4833 | -0.3252 | 0.1581 |  | |
| 57 | PgR009X | 3430001 | 3440000 | 0.4332 | 0.2954 | 0.0698 | 0.3558 | -0.1296 | -0.4854 | SSBP2 | |
| 58 | PgR001X | 1420001 | 1430000 | 0.4301 | 0.3574 | 0.1094 | 0.9423 | 0.3177 | -0.6246 | GLY1 | |
| 59 | PgB02X | 320001 | 330000 | 0.4264 | 0.0098 | 0.3147 | 1.343 | 0.3923 | -0.9507 |  | |
| 60 | PgB02X | 2940001 | 2950000 | 0.4263 | 0.2489 | 0.0318 | 0.7795 | 0.4177 | -0.3618 |  | |
| 61 | PgR020 | 1930001 | 1940000 | 0.4256 | -0.0284 | 0.5657 | 0.8096 | 0.3003 | -0.5093 |  | |
| 62 | PgR062 | 390001 | 400000 | 0.4191 | 0.4428 | 0.0093 | 0.8223 | 2.0612 | 1.2389 | HK2 | |
| 63 | PgR004 | 4100001 | 4110000 | 0.4191 | 0.3273 | 0.0162 | -0.431 | -0.2922 | 0.1387 |  | |
| 64 | PgR010 | 1220001 | 1230000 | 0.4171 | -0.0623 | 0.4697 | 0.5085 | 0.4659 | -0.0426 |  | |
| 65 | PgR020 | 1770001 | 1780000 | 0.4164 | 0.0371 | 0.1816 | -0.6591 | -0.1046 | 0.5545 | Mto1 | |
| 66 | PgR023 | 490001 | 500000 | 0.4152 | 0.2444 | 0.0916 | 4.0672 | 2.039 | -2.0281 |  | |
| 67 | PgB02X | 1230001 | 1240000 | 0.4152 | 0.3194 | -0.0456 | 1.7848 | 1.3232 | -0.4616 |  | |
| 68 | PgR035X | 1460001 | 1470000 | 0.4152 | 0.3747 | -0.1076 | 1.2012 | 1.0814 | -0.1198 | Gls2 | |
| 69 | PgR009X | 1990001 | 2000000 | 0.4143 | 0.1635 | 0.2804 | 0.4593 | -0.4405 | -0.8999 | Snd1 | |
| 70 | PgR024 | 1500001 | 1510000 | 0.4112 | 0.1405 | 0.0766 | 0.181 | 0.1489 | -0.032 | Abat | |
| 71 | PgR009X | 1500001 | 1510000 | 0.409 | 0.1937 | 0.0816 | 0.1824 | -0.6085 | -0.7909 |  | |
| 72 | PgR050X | 1090001 | 1100000 | 0.4081 | 0.3268 | -0.054 | 2.2878 | 2.5722 | 0.2843 |  | |
| 73 | PgR035X | 520001 | 530000 | 0.4067 | 0.128 | 0.0849 | 0.9689 | -0.133 | -1.102 | dus4 | |
| 74 | PgR028 | 170001 | 180000 | 0.4 | 0.1894 | 0.1376 | -0.3015 | -0.3333 | -0.0318 | Rab28 | |
| 75 | PgR052 | 1080001 | 1090000 | 0.3977 | 0.2993 | -0.0275 | -0.2826 | -0.9411 | -0.6585 |  | |
| 76 | PgR032X | 750001 | 760000 | 0.397 | 0.2099 | -0.0324 | -0.1067 | -0.512 | -0.4053 | Col6a4 | |
| 77 | PgR040 | 440001 | 450000 | 0.3924 | 0.3307 | -0.0265 | 0.0576 | 0.4177 | 0.3602 | CAT2 | |
| 78 | PgR009X | 1440001 | 1450000 | 0.3904 | 0.3711 | 0.0803 | 0.1115 | -0.9474 | -1.0589 | nas-36，TLL1 | |
| 79 | PgB07 | 160001 | 170000 | 0.3903 | 0.2478 | 0.04 | 2.6927 | 2.6492 | -0.0435 |  | |
| 80 | PgB12X | 1260001 | 1270000 | 0.3903 | 0.4377 | -0.0998 | -0.2056 | -0.703 | -0.4974 |  | |
| 81 | PgR002 | 600001 | 610000 | 0.3892 | 0.1292 | 0.1236 | -0.5981 | -0.4967 | 0.1014 | ADAMTS6 | |
| 82 | PgR052 | 1120001 | 1130000 | 0.3884 | 0.2425 | -0.0837 | -0.0675 | -0.6626 | -0.5951 | RAB38 | |
| 83 | PgR051X | 540001 | 550000 | 0.3862 | 0.0203 | 0.1325 | 0.7459 | -0.0814 | -0.8274 | lem-3 | |
| 84 | PgR028 | 180001 | 190000 | 0.3862 | 0.2062 | 0.1276 | -0.2621 | -0.2197 | 0.0424 | FOXP2 | |
| 85 | PgR063X | 90001 | 100000 | 0.3815 | 0.3009 | -0.0755 | 1.8505 | 0.9368 | -0.9137 | nop2，foxg1 | |
| 86 | PgB07 | 150001 | 160000 | 0.3799 | 0.2396 | 0.06.65 | 3.153 | 3.4065 | 0.2535 |  | |
| 87 | PgB02X | 2910001 | 2920000 | 0.3781 | 0.1993 | 0.0673 | 1.0475 | 0.5919 | -0.4556 |  | |
| 88 | PgR028 | 1520001 | 1530000 | 0.3758 | 0.5723 | 0.0154 | -0.3074 | 0.6108 | 0.9182 |  | |
| 89 | PgR010 | 2830001 | 2840000 | 0.3753 | 0.1523 | 0.0419 | 0.8499 | 0.9656 | 0.1157 | UMOD | |
| 90 | PgR040 | 210001 | 220000 | 0.3744 | 0.0681 | 0.1228 | -0.5397 | -0.5082 | 0.0315 | R3hdm4 | |
| 91 | PgR035X | 500001 | 510000 | 0.3736 | 0.1177 | 0.038 | 0.4049 | -0.3625 | -0.7674 |  | |
| 92 | PgR029 | 820001 | 830000 | 0.3732 | 0.452 | -0.0174 | -0.0998 | 0.5685 | 0.6683 | Fer | |
| 93 | PgB11 | 1270001 | 1280000 | 0.3716 | 0.2902 | -0.0319 | 2.133 | 3.0858 | 0.9528 |  | |
| 94 | PgR023 | 1690001 | 1700000 | 0.3714 | 0.5576 | 0.0664 | -0.2191 | 0.0549 | 0.2739 | Qki | |
| 95 | PgR011 | 1610001 | 1620000 | 0.3701 | 0.2414 | 0.1012 | -0.6603 | 0.0218 | 0.6822 |  | |
| 96 | PgB04 | 620001 | 630000 | 0.3693 | 0.2399 | 0.1097 | 1.0757 | 0.4818 | -0.5938 |  | |
| 97 | PgR009X | 2570001 | 2580000 | 0.3692 | 0.0037 | 0.1336 | 0.8624 | 0.2917 | -0.5708 |  | |
| 98 | PgR001X | 630001 | 640000 | 0.3689 | 0.1489 | 0.0011 | 0.6627 | 0.2676 | -0.3951 |  | |
| 99 | PgR023 | 100001 | 110000 | 0.3687 | 0.3876 | -0.1086 | -0.18 | -0.6179 | -0.4379 | nAChRalpha2 | |
| 100 | PgR007 | 320001 | 330000 | 0.3681 | 0.4682 | 0.0234 | 0.5874 | 1.458 | 0.8706 | Lgr4 | |

**Table S7** GO enrichment (Top 20) of iHS significant selection sites in PEc and PEz&PEa clades.

| No. | TermID | Name | Frequency |
| --- | --- | --- | --- |
| PEc |  |  |  |
| 1 | GO:0008152 | metabolic process | 47.116 |
| 2 | GO:0010468 | regulation of gene expression | 15.97 |
| 3 | GO:0006355 | regulation of transcription DNA-templated | 11.07 |
| 4 | GO:0007186 | G protein-coupled receptor signaling pathway | 7.434 |
| 5 | GO:0006396 | RNA processing | 5.164 |
| 6 | GO:0016192 | vesicle-mediated transport | 4.464 |
| 7 | GO:0044255 | cellular lipid metabolic process | 4.252 |
| 8 | GO:0007399 | nervous system development | 3.584 |
| 9 | GO:0006886 | intracellular protein transport | 3.521 |
| 10 | GO:0051603 | proteolysis involved in cellular protein catabolic process | 3.043 |
| 11 | GO:0010629 | negative regulation of gene expression | 2.715 |
| 12 | GO:0006511 | ubiquitin-dependent protein catabolic process | 2.471 |
| 13 | GO:0006397 | mRNA processing | 2.333 |
| 14 | GO:0042254 | ribosome biogenesis | 2.121 |
| 15 | GO:0006281 | DNA repair | 1.951 |
| 16 | GO:0006631 | fatty acid metabolic process | 1.516 |
| 17 | GO:0000398 | mRNA splicing via spliceosome | 1.326 |
| 18 | GO:0006486 | protein glycosylation | 1.304 |
| 19 | GO:0006351 | transcription DNA-templated | 1.22 |
| 20 | GO:0034976 | response to endoplasmic reticulum stress | 1.05 |
| PEz & PEa | |  |  |
| 1 | GO:0008152 | metabolic process | 47.116 |
| 2 | GO:0010468 | regulation of gene expression | 15.97 |
| 3 | GO:0006355 | regulation of transcription DNA-templated | 11.07 |
| 4 | GO:0007186 | G protein-coupled receptor signaling pathway | 7.434 |
| 5 | GO:0006396 | RNA processing | 5.164 |
| 6 | GO:0016192 | vesicle-mediated transport | 4.464 |
| 7 | GO:0044255 | cellular lipid metabolic process | 4.252 |
| 8 | GO:0007399 | nervous system development | 3.584 |
| 9 | GO:0006886 | intracellular protein transport | 3.521 |
| 10 | GO:0051603 | proteolysis involved in cellular protein catabolic process | 3.043 |
| 11 | GO:0010629 | negative regulation of gene expression | 2.715 |
| 12 | GO:0006511 | ubiquitin-dependent protein catabolic process | 2.471 |
| 13 | GO:0006397 | mRNA processing | 2.333 |
| 14 | GO:0042254 | ribosome biogenesis | 2.121 |
| 15 | GO:0006281 | DNA repair | 1.951 |
| 16 | GO:0006631 | fatty acid metabolic process | 1.516 |
| 17 | GO:0000398 | mRNA splicing via spliceosome | 1.326 |
| 18 | GO:0006486 | protein glycosylation | 1.304 |
| 19 | GO:0006351 | transcription DNA-templated | 1.22 |
| 20 | GO:0034976 | response to endoplasmic reticulum stress | 1.05 |

**Table S8** KEGG enrichment of XP-EHH significant selection sites in PEc and PEz&PEa clades

| Pathway | | | Sample1 | | Sample2 | Pvalue | | | Qvalue | | | Pathway ID |
| --- | --- | --- | --- | --- | --- | --- | --- | --- | --- | --- | --- | --- |
| PEc | | |  | |  | | |  | | |  | |
| Fc gamma R-mediated phagocytosis | | | 9 | | 60 | 0.0022 | | | 0.4685 | | | ko04666 |
| RNA polymerase | | | 7 | | 52 | 0.0121 | | | 0.8222 | | | ko03020 |
| Butanoate metabolism | | | 5 | | 32 | 0.0178 | | | 0.8222 | | | ko00650 |
| Cytosolic DNA-sensing pathway | | | 5 | | 35 | 0.0256 | | | 0.8222 | | | ko04623 |
| Glutamatergic synapse | | | 8 | | 74 | 0.0262 | | | 0.8222 | | | ko04724 |
| Propanoate metabolism | | | 6 | | 48 | 0.0274 | | | 0.8222 | | | ko00640 |
| Sphingolipid metabolism | | | 6 | | 48 | 0.0274 | | | 0.8222 | | | ko00600 |
| Lysine degradation | | | 7 | | 63 | 0.0319 | | | 0.8222 | | | ko00310 |
| Synthesis and degradation of ketone bodies | | | 3 | | 16 | 0.0396 | | | 0.8222 | | | ko00072 |
| ABC transporters | | | 5 | | 40 | 0.0426 | | | 0.8222 | | | ko02010 |
| PEz & PEa |  |  | |  | | |  | | |  | | |
| RNA transport | | | 21 | | 160 | 0.0066 | | | 0.9989 | | | ko03013 |
| Selenocompound metabolism | | | 4 | | 15 | 0.0206 | | | 0.9989 | | | ko00450 |
| Mismatch repair | | | 5 | | 25 | 0.0329 | | | 0.9989 | | | ko03430 |
| Inositol phosphate metabolism | | | 8 | | 54 | 0.0423 | | | 0.9989 | | | ko00562 |
| Nicotinate and nicotinamide metabolism | | | 5 | | 31 | 0.0735 | | | 0.9989 | | | ko00760 |
| Pentose phosphate pathway | | | 5 | | 31 | 0.0735 | | | 0.9989 | | | ko00030 |
| Pantothenate and CoA biosynthesis | | | 4 | | 23 | 0.0841 | | | 0.9989 | | | ko00770 |
| Aminoacyl-tRNA biosynthesis | | | 8 | | 64 | 0.0958 | | | 0.9989 | | | ko00970 |
| mRNA surveillance pathway | | | 16 | | 154 | 0.1007 | | | 0.9989 | | | ko03015 |
| Basal transcription factors | | | 6 | | 47 | 0.1284 | | | 0.9989 | | | ko03022 |

**Supplementary Figures**


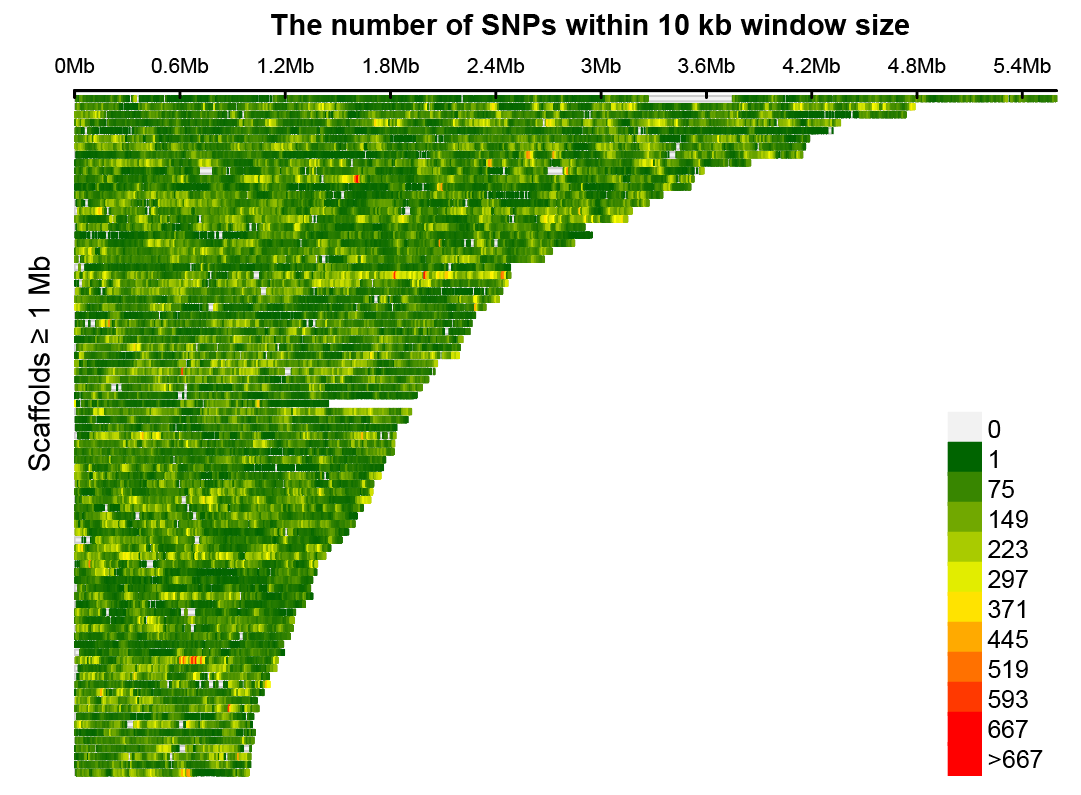


**Figure S1** SNP density of all samples.


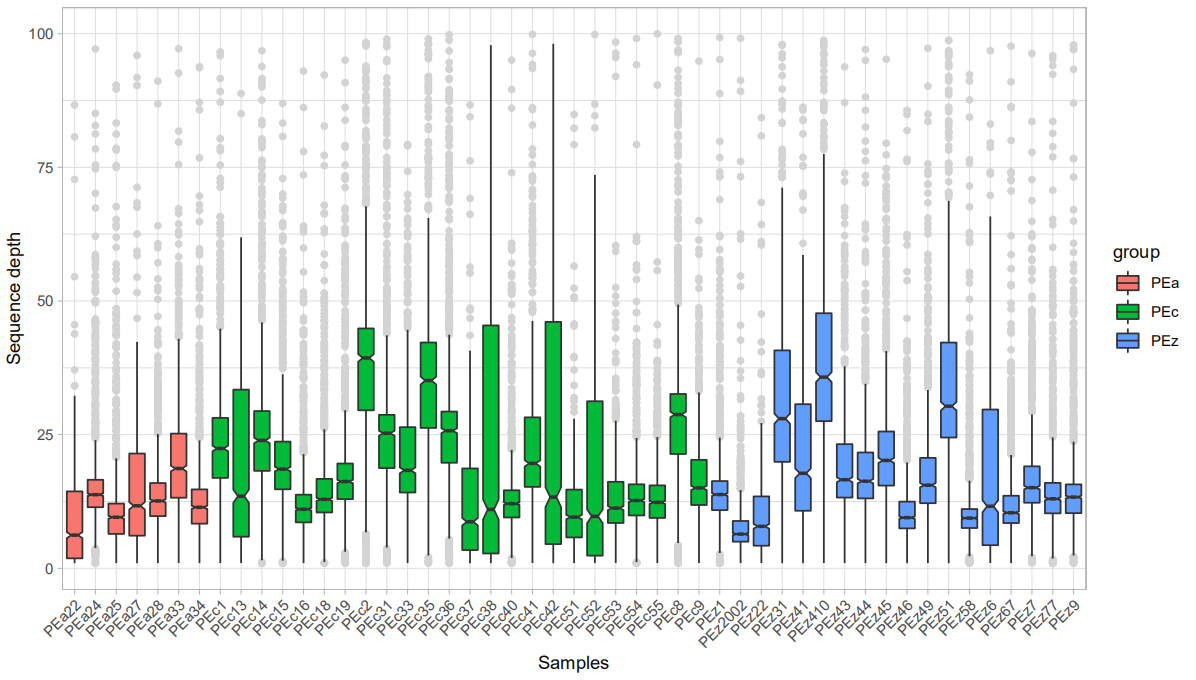


**Figure S2** Sequencing depth of PEa, PEc and PEz populations.


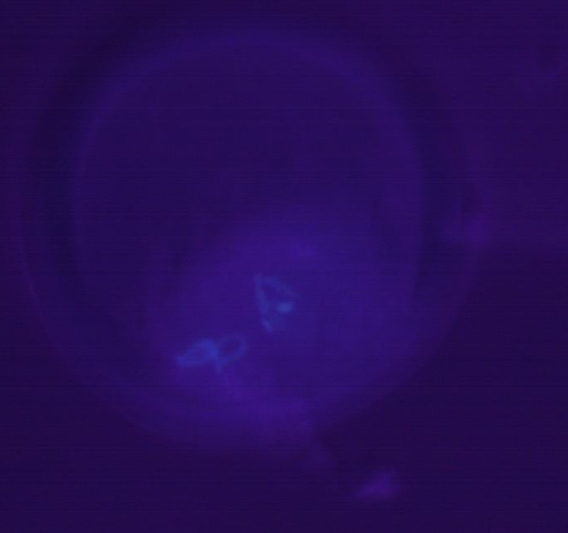


**Figure S3** Karyotyping of *P. univalens*.


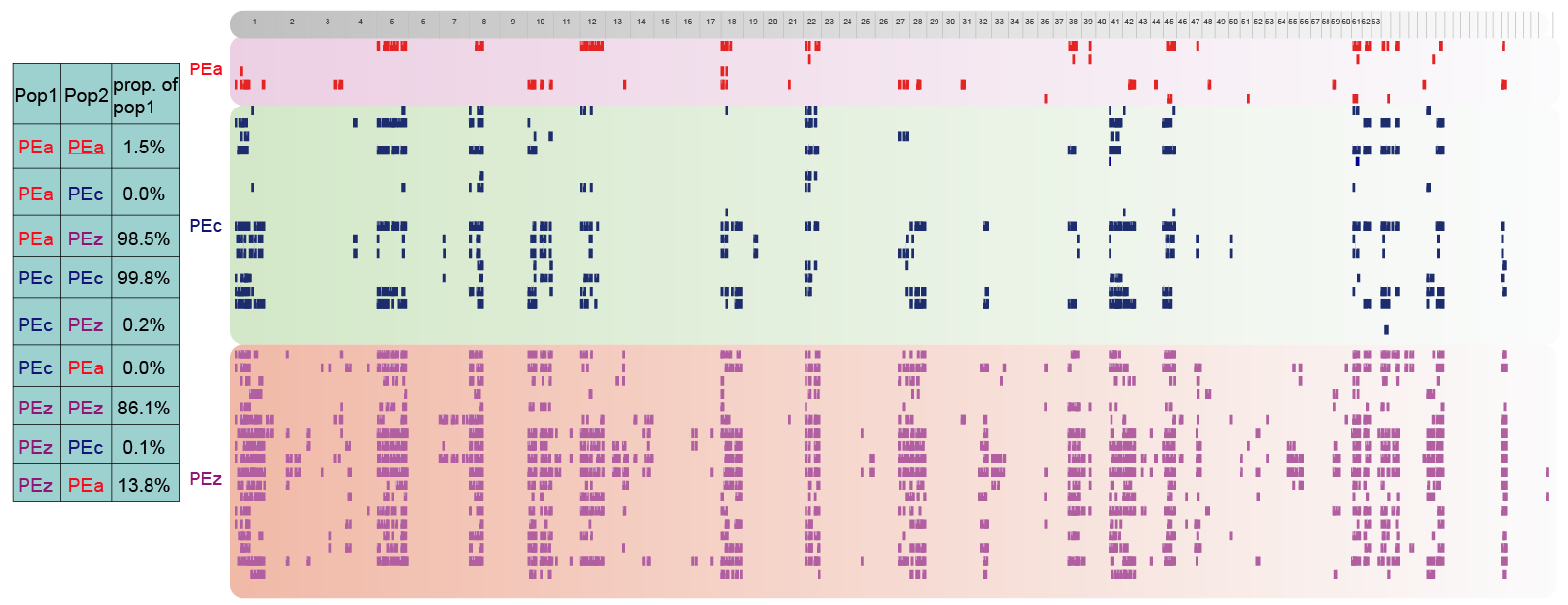


**Figure S4** Shared identity-by-descent (IBD) regions among PEa, PEc and PEz populations.


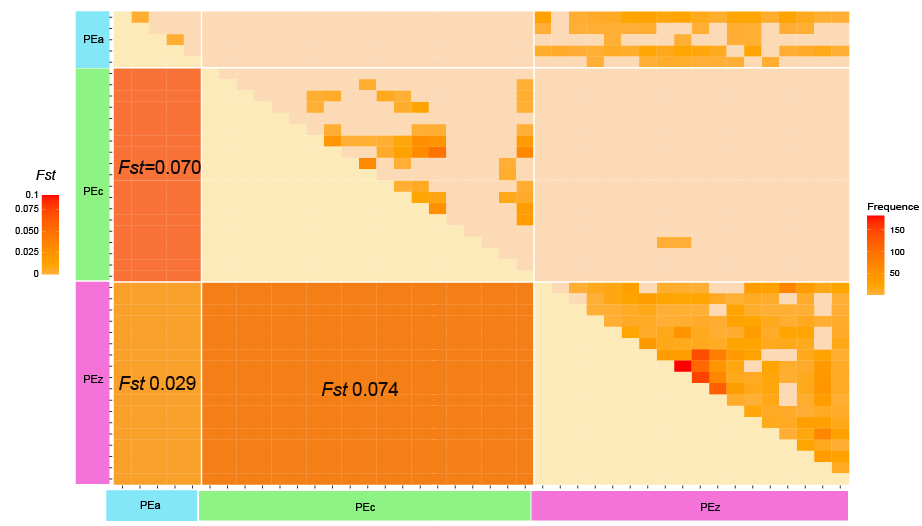


**Figure S5** The shared IBD and paired *Fst* values of the three populations.


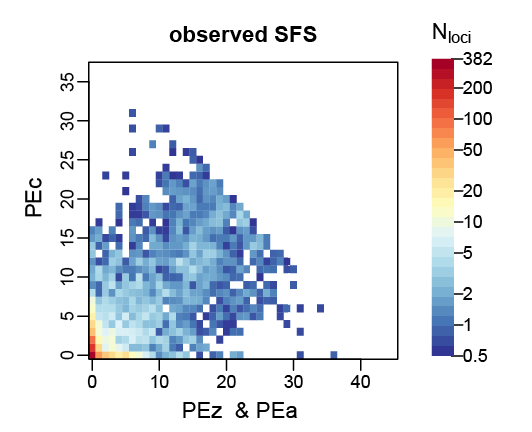


**Figure** **S6** Observed SFS between PEc and PEz & PEa clades.


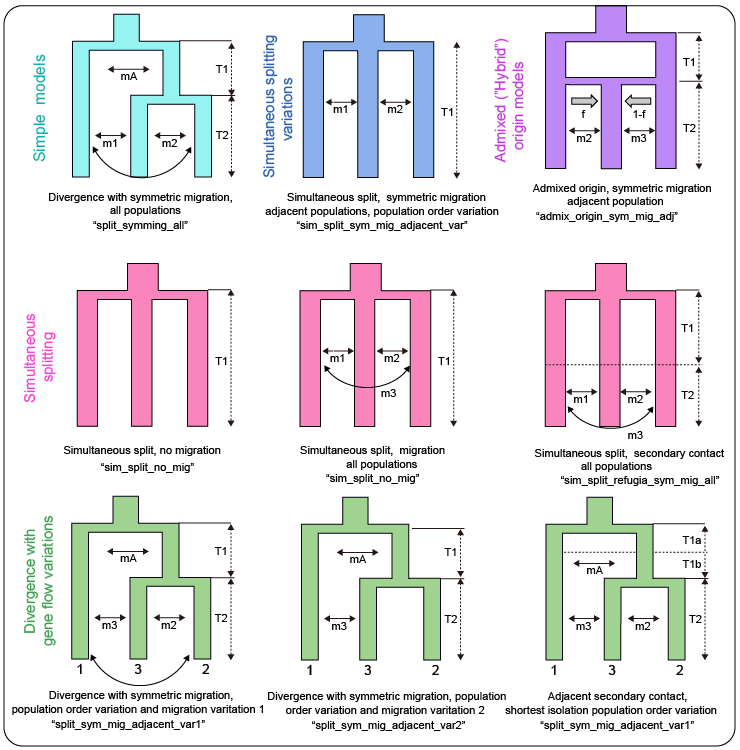


**Figure S7** 3D divergence model used in δaδi.


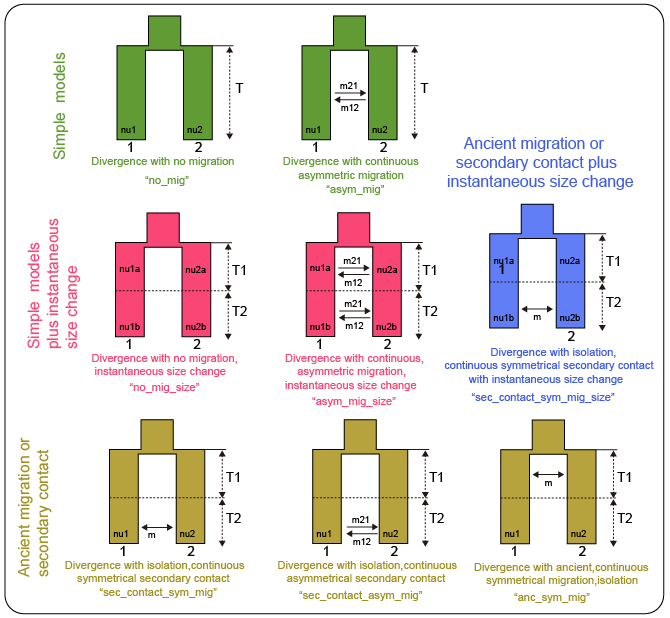


**Figure S8** 2D divergence model used in δaδi.


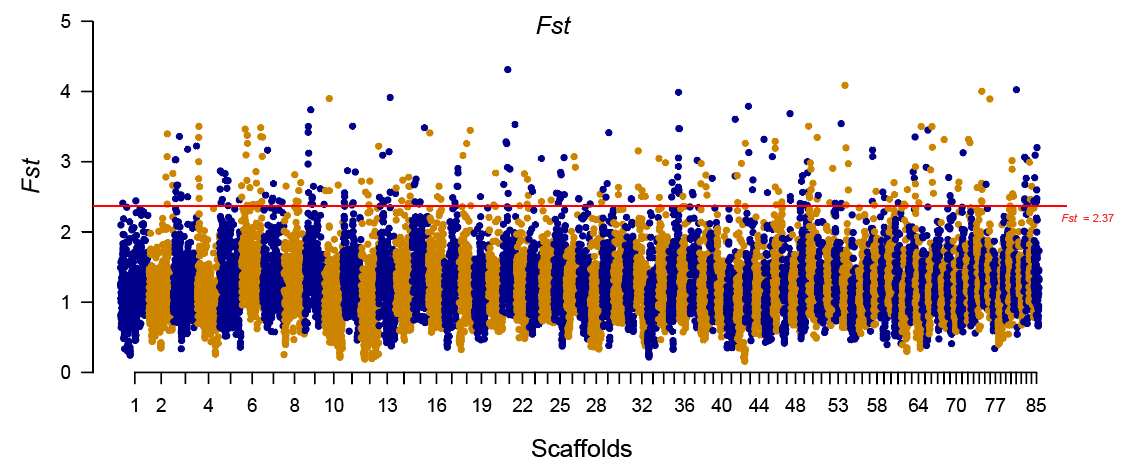


**Figure S9** *Fst* distribution of PEc vs PEa & PEz.


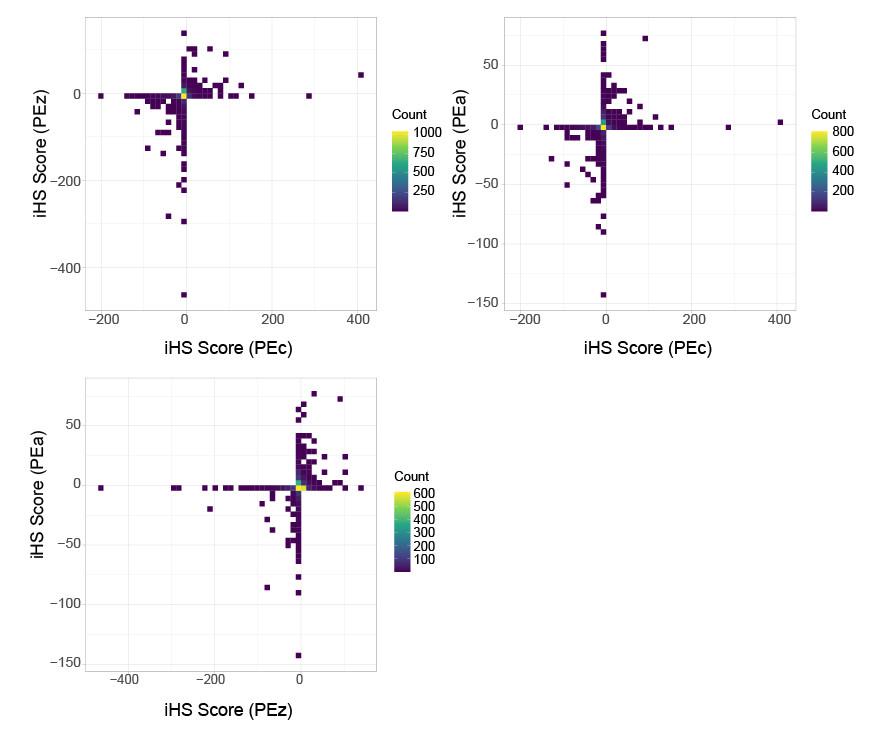


**Figure S10** The iHS score distribution of PEc, PEa and PEz populations.


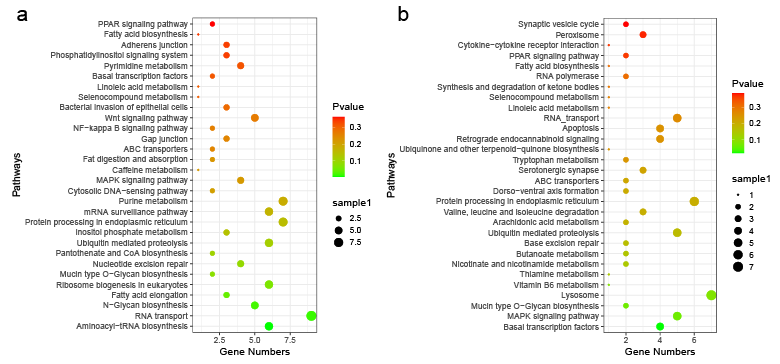


**Figure S11** KEGG enrichment of iHS significant selection locis of PEc and PEa & PEz respectively.


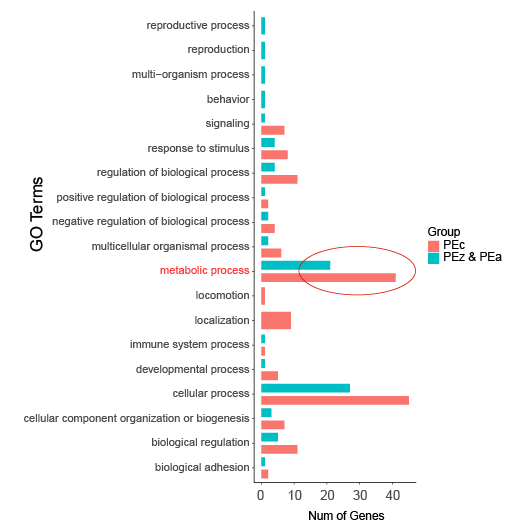


**Figure S12** GO enrichment of XP-EHH analysis of PEa and PEc & PEz respectively.


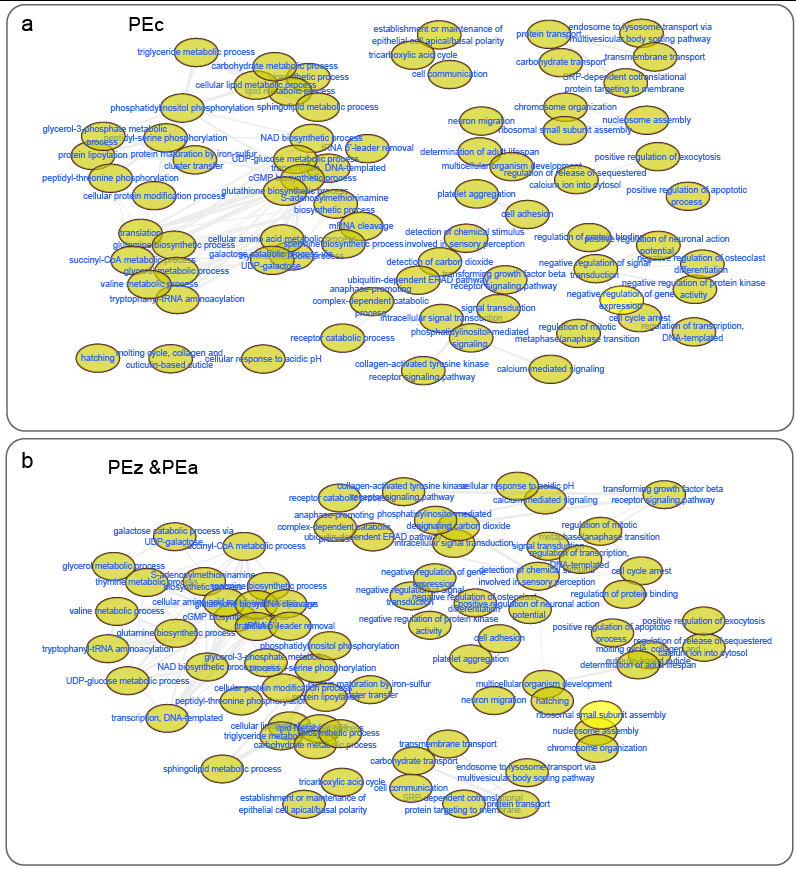


**Figure S13** Network diagram of GO function enrichment obtained by XP-EHH analysis of PEa and PEc & PEz clades.


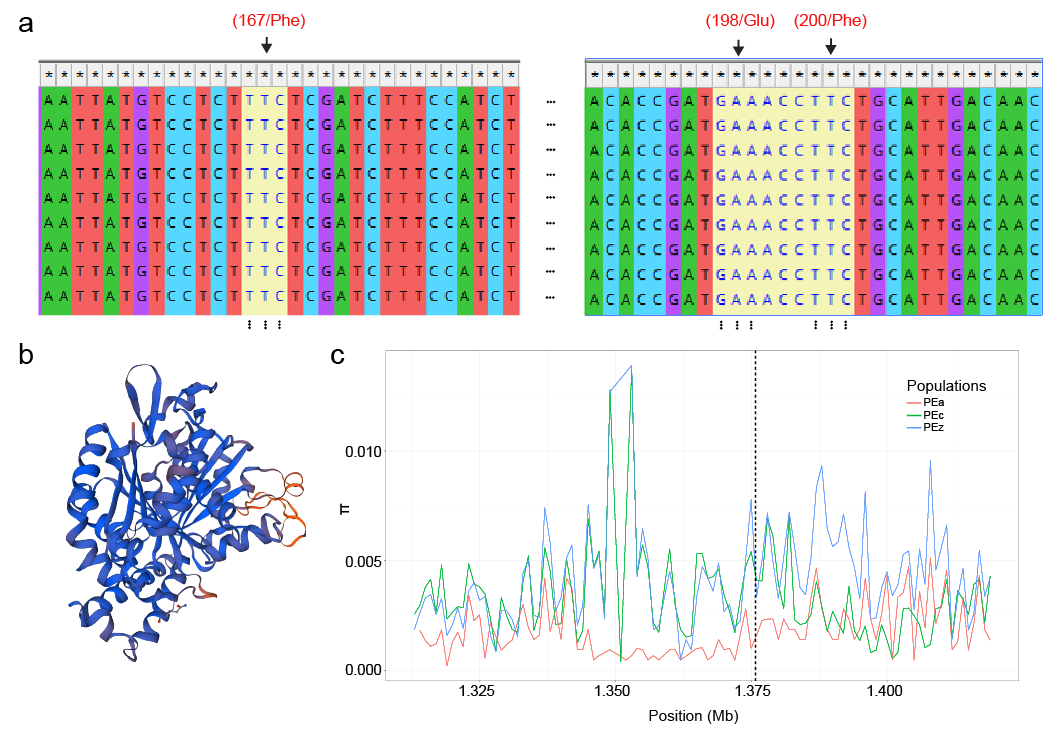


**Figure S14** Detection of mutations in three drug-resistant sites of β-tubulin. (**a**) No drug-resistant base mutations were detected at positions 167/Phe, 198/Glu and 200/Phe in all individuals. (**b**) The 3D structure of β-tubulin. (**c**) The *π* distribution around β-tubulin region of the three populations.


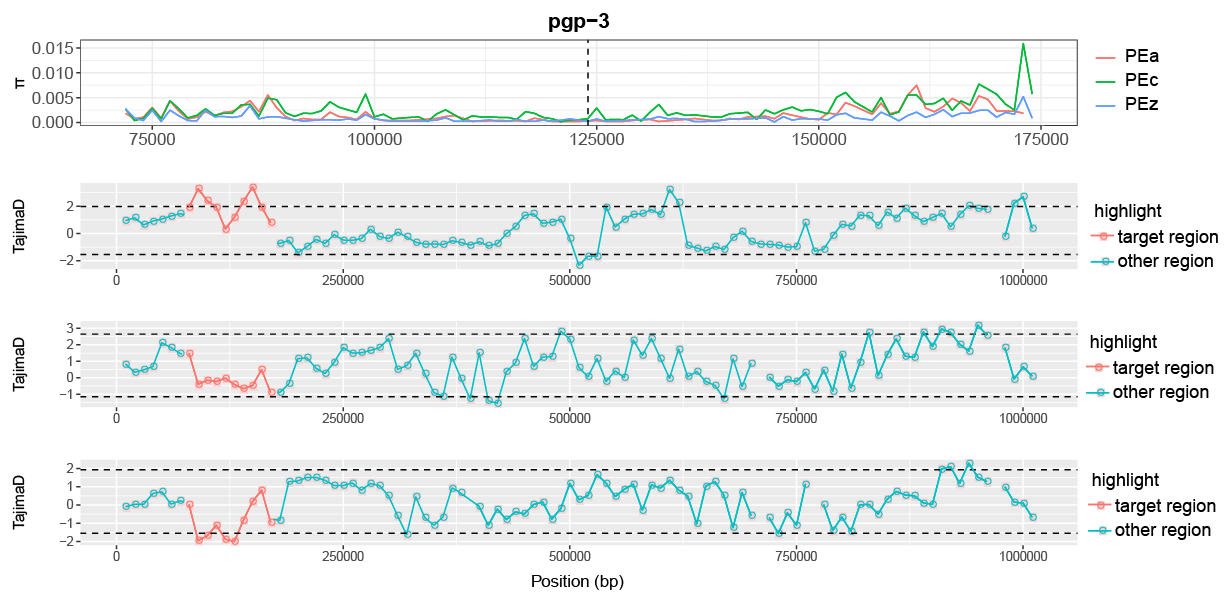


**Figure S15** The *π* distribution and Tajima’D distribution in the *pgp-3* regions of PEc, PEz and PEa populations.


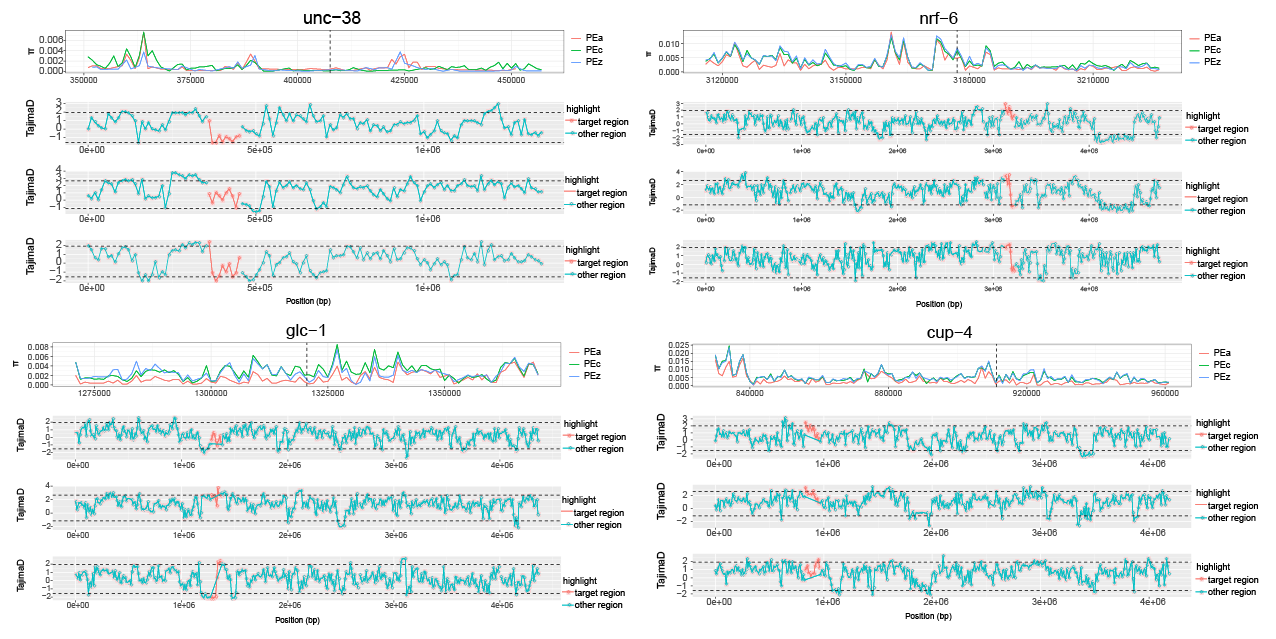


**Figure S16** The *π* distribution and *tajima’D* distribution in the *unc-38*, *nrf-6*, *glc-1* and *cup-4* regions of PEc, PEz and PEa populations.


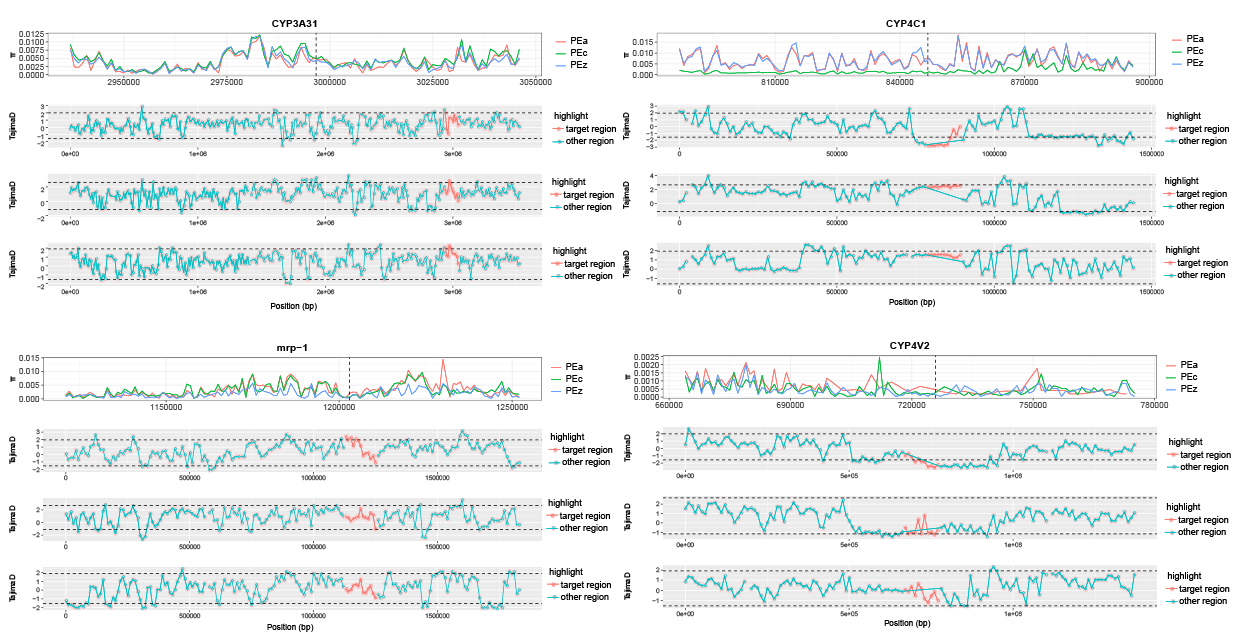


**Figure S17** The *π* distribution and tajima’D distribution in the *CYP3A31*, *CYP4C1*, *mrp-1* and *CYP4V2* regions of PEc, PEz and PEa populations.
